# Calcium Signaling in Vertebrate Development and Its Role in Disease

**DOI:** 10.3390/ijms19113390

**Published:** 2018-10-30

**Authors:** Sudip Paudel, Regan Sindelar, Margaret Saha

**Affiliations:** College of William and Mary, Williamsburg, VA 23187, USA; spaudel@email.wm.edu (S.P.); rssindelar@email.wm.edu (R.S.)

**Keywords:** calcium, development, embryo, human disease, animal model, *Xenopus*, zebrafish, mouse

## Abstract

Accumulating evidence over the past three decades suggests that altered calcium signaling during development may be a major driving force for adult pathophysiological events. Well over a hundred human genes encode proteins that are specifically dedicated to calcium homeostasis and calcium signaling, and the majority of these are expressed during embryonic development. Recent advances in molecular techniques have identified impaired calcium signaling during development due to either mutations or dysregulation of these proteins. This impaired signaling has been implicated in various human diseases ranging from cardiac malformations to epilepsy. Although the molecular basis of these and other diseases have been well studied in adult systems, the potential developmental origins of such diseases are less well characterized. In this review, we will discuss the recent evidence that examines different patterns of calcium activity during early development, as well as potential medical conditions associated with its dysregulation. Studies performed using various model organisms, including zebrafish, *Xenopus*, and mouse, have underscored the critical role of calcium activity in infertility, abortive pregnancy, developmental defects, and a range of diseases which manifest later in life. Understanding the underlying mechanisms by which calcium regulates these diverse developmental processes remains a challenge; however, this knowledge will potentially enable calcium signaling to be used as a therapeutic target in regenerative and personalized medicine.

## 1. Introduction

Calcium is an ancient and ubiquitous signaling ion involved in a wide array of physiological processes throughout the entire lifespan of the individual, from fertilization through senescence. Given its central position in virtually every cellular process and tissue type, it is not surprising that its dysregulation leads to a wide spectrum of pathophysiological conditions. These conditions include, but are not limited to, infertility, miscarriage, developmental defects, neuropathic pain, epilepsy and seizures [1].

Under normal conditions, the basal intracellular concentration of calcium ion (Ca^2+^) is maintained at very low levels compared to the extracellular spaces by a number of proteins, including pumps and transporters that have Ca^2+^ binding ability. Calcium pumps, using ATP, remove Ca^2+^ from the cytosol and help cells maintain roughly hundred to thousand-fold concentration gradients between intracellular and extracellular environments. Ca^2+^-ATPases are an example of these pumps, which are localized in various cellular organelles; for example, P2A ATPase is localized in the endoplasmic reticulum, P2B ATPase is localized in the plasma membrane, and P2C ATPase is localized in the Golgi apparatus [2,3,4]. In addition, Ca^2+^ enters the cytosol passively from the extracellular matrix, neighboring cells, and cytoplasmic Ca^2+^ stores; the opening and closing of these channels is driven by both chemical and electrical gradients. Cells create these gradients in order to use calcium as a signaling molecule [3]. During calcium-mediated signaling events, Ca^2+^ permeant channels open transiently allowing spontaneous influx of Ca^2+^ into the cytosol. This influx of Ca^2+^ increases intracellular concentration of Ca^2+^, and is often referred to generically as a calcium transient [5,6,7]. The short transient fluctuation in cytoplasmic Ca^2+^ within a given (typically isolated) cell due to these influxes are often defined as calcium spikes [8]. When a calcium spike originates from one or a few cells and then propagates along several other neighboring cells via gap junctions or paracrine signaling, it is called an intercellular wave [5,8,9,10]. The change in concentration of cytoplasmic Ca^2+^ is sensed by a number of calcium sensing proteins with calcium binding affinities ranging from hundreds to million-fold [11]. These calcium sensing proteins transduce calcium signals, decoding spatial and temporal changes in amplitude, duration and localization of calcium activity [7,11,12]_._

In turn, these signaling cascades drive a wide array of physiological responses throughout the life of the organism from fertilization onwards. While the role of calcium and its associated pathologies have been intensively studied in the adult organism, less attention has been focused on the role of calcium and its relationship to disease during embryonic development. Given the importance of calcium activity as a regulator of embryonic development from fertilization through organogenesis, dysregulation of these calcium dynamics, due to either mutations or epigenetic developmental dysregulation of some element or component of the calcium signaling process, results in various diseases and disorders (Supplementary Table S1). For example, Timothy syndrome is caused by constitutive opening of voltage gated calcium channel CaV1.2 due to mutations in the *cacna1c* gene. This gene is expressed embryonically and the syndrome is characterized by syndactyly, intellectual disability, congenital heart defects, distinctive facial features and developmental delay. Similarly, mutations in the gene ATP2A1 (sarco(endo)plasmic reticulum calcium-ATPase 1 (SERCA1)) on chromosome 16p11 result in Brody myopathy, which is characterized by a decrease or loss of sarcoplasmic reticulum Ca^2+^-ATPase activity and problems with muscle contraction [13]. While some of these mutations and dysregulated processes are embryonic lethal, many manifest their effects at birth, and others may not show symptoms until later in life due to their indirect effects within a complex genetic network [14].

Calcium activity during development is varied and complex with embryos exhibiting different patterns of spikes and waves. Animal model studies during early stages of development have provided a broad understanding of human developmental defects and diseases related to the dysregulation of calcium activity. In this review, we will provide an overview of the current state of knowledge regarding the role of calcium activity in embryonic and fetal development and disease. Given the obvious challenges of studying calcium activity in human embryonic development, much of the information we will discuss derives from model systems, particularly frogs, fish, and mice. We will discuss each stage of development from fertilization through organogenesis chronologically. Each section will begin with a brief overview of the key developmental events that occur during that particular stage and then proceed to analyze the role of calcium in those processes, including how dysregulation of calcium dynamics can, and does, lead to disease.

## 2. Calcium Activity during Development and Its Role in Disease

### 2.1. Fertilization and Egg Activation

Fertilization is the process by which DNA of the sperm and egg unite to give rise to a new diploid organism. Sperm entry then triggers the oocyte to transition into a developing embryo in a process known as egg activation. Egg activation is characterized by the occurrence of a number of sequential events in the oocyte during fertilization: recruitment of maternal mRNA and formation of polysomes, completion of meiosis, modification of the plasma membrane and zona pellucida in order to prevent polyspermy, cortical granule exocytosis, formation of male and female proneuclei, and syngamy, the fusion of two genomes [15,16]. While species differences exist, the process of egg activation is a relatively conserved mechanism that is mediated and coordinated by calcium; failure in any step of this process typically results in infertility.

The importance of calcium activity in the process of fertilization and egg activation cannot be underestimated. Fertilization initiates elevations of intracellular Ca^2+^ concentration in all vertebrate oocytes studied to date [17]. These elevations are initiated from the site of sperm-egg fusion, and are caused by transient influxes of Ca^2+^ from both the extracellular milieu and intracellular calcium stores. The patterns of these influxes do vary somewhat across species. For example, the oocytes from some lower vertebrates such as *Xenopus* and zebrafish achieve this elevation via a single calcium transient, while mammalian oocytes exhibit an initial transient increase within a few minutes of the sperm binding to the egg surface, followed by subsequent oscillations in cytoplasmic Ca^2+^ concentration at 20 to 30 min intervals [17,18,19]. This calcium activity was visualized for the first time in a mammalian egg by imaging zona-free mouse oocytes using aequorin during in vitro fertilization [20]. Similar calcium behavior was observed in mouse and human oocytes during in vitro fertilization and intracytoplasmic sperm injection using aequorin or various other calcium sensitive dyes [21,22]. Inhibition of this calcium activity results in fertilization failure. For example, when extracellular Ca^2+^ was restricted from entering the oocyte cytoplasm using bivalent cation chelators such as BAPTA or EGTA, calcium insulators such as gadolinium, or a Ca^2+^ free culture medium, *Xenopus* oocytes failed to develop normal cleavage furrows, and mouse oocytes failed to resume meiosis during the egg activation process [18,23,24]. Similarly, wild type sperm was unable to activate zona-free mouse eggs lacking a gene (cacna1h^−/−^) that codes for a T-type voltage gated calcium channel, which mediates extracellular calcium entry [25]. Pharmacological inhibition of this channel, using mibefradil or pimozide, also resulted in a phenotype similar to cacna1h^−/−^ following in vitro fertilization [25]. These observations suggest that impaired entry of calcium from extracellular matrix to the oocyte cytoplasm may result in fertilization failure and lead to infertility.

Sperm specific phospholipase C, such as phospholipase C isozyme zeta, on its own is sufficient to trigger calcium release from intracellular calcium stores via channels, including STIM1, in order to accomplish egg activation in different species [17]. Mouse eggs injected with sperm-supernatants treated with an anti-phospholipase C zeta antibody failed to show Ca^2+^ elevation and subsequent egg activation events. However, when the eggs were subsequently microinjected with only sperm specific phospholipase C zeta, normal elevation of Ca^2+^ was observed and egg activation was rescued [26,27]. These observations have been further strengthened by recent gene editing technology. For example, intracytoplasmic sperm injection or in vivo fertilization with phospholipase C zeta knockout sperm failed to trigger egg activation in wild type mouse eggs, but subsequent injection of wild type phospholipase C zeta-RNA successfully rescued egg activation and zygotic development [28]. Various measures, including histological analysis, sperm viability, sperm motility and hyperactivity assays, showed that phospholipase C zeta gene knockout using CRISPR/Cas9 results in no defects in spermatogenesis [28]. Similarly, the knock down of STIM1 from pig oocytes using STIM1 specific siRNA resulted in failure to generate Ca^2+^ oscillations, decreased oocyte survival rate, and decreased efficiency of cleavage and blastocyst development [29]. Taken together, these observations suggest that impaired function of sperm-specific phospholipase C zeta also results in impaired egg activation leading to mammalian infertility. Inhibition of these Ca^2+^ elevations results in failure to initiate and complete functional fertilization in humans and other model organisms studied to date, including mice, *Xenopus* and zebrafish [16,30,31].

Recent molecular advances, including genomics, transcriptomics, proteomics, gene editing technology, and imaging technology, are opening new avenues of exploration for the potential therapeutic use of calcium in infertility treatments. For instance, assisted oocyte activation using mechanical, chemical, or electrical artificial triggers that cause calcium influx into the oocyte cytoplasm have been shown to trigger egg activation [30]. Therefore, manipulation of calcium activity could serve as a potential therapy for severe infertility associated with egg activation failure.

### 2.2. Cleavage and Blastula Stages

Following egg activation, vertebrate embryos undergo a rapid series of cell divisions leading to a relatively unstructured and undifferentiated mass of fluid-filled cells termed the blastula. It is during this stage that the maternal to zygotic transition occurs in which the zygotic genome is activated, an event that happens very early in mammals. It is also during this stage of mammalian development that the first important developmental lineage decisions occur, namely the determination of the inner cell mass that will give rise to the embryo proper and the trophoblast that will eventually give rise to the extraembryonic tissues. The blastula stage occurs during the first few weeks of embryonic development in humans; developmental defects during this stage of development affect germ layer formation and morphogenetic movements and will typically result in a non-viable embryo [32].

Calcium plays a key role in the regulation of the cell divisions during this early stage of development. During the initial cleavage stages, the embryo exhibits slowly moving intracellular waves of calcium. When imaging transgenic zebrafish that express genetically encoded calcium indicators, including cameleon YC2.60 and GCaMP6 driven by promoters of constitutive genes such as hspa8 and βactin, these waves were localized exclusively to the cleavage furrow [19,33]. Immunocytochemistry assays in zebrafish embryos have shown that this furrow-associated calcium activity is mediated by store-operated Ca^2+^ entry channels, including STIM1 and ORAI1 [34,35]. Furrow-associated waves have been shown to remodel microtubules into a furrow microtubule array that is required for cytokinesis in zebrafish. The furrow microtubule array is analogous to the mammalian midbody during the final stage of cytokinesis. The furrow microtubule array remodeling process is mediated by *nebel*, a cytokinesis regulator gene. When *nebel* mutant embryos were imaged using Oregon-Green BAPTA dextran, they not only showed reductions in both amplitude and frequency of slowly moving intracellular waves of calcium, but also failed to undergo cleavage. However, when calcium activity was enhanced by injecting IP3, NAADP, and CaCl_2_ solution into *nebel* mutant eggs, furrow microtubule array formation was increased and the eggs progressed towards pseudocleavage [36]. These observations underscore the importance of calcium during the first divisions of zygotic development.

As embryos develop, furrow-associated Ca^2+^ activity accompanies successive cleavages until the 64-cell stage and then becomes undetectable, at least in zebrafish [37]. These results were consistent with findings from previous studies in wild type zebrafish [38]. During later blastula stages, namely the 128 cell-stage in zebrafish, both forms of calcium activity (spikes and waves) are observed. During a calcium spike, cytoplasmic calcium activity spreads over a whole cell, whereas in a calcium wave, it propagates across a group of contiguous or nearly contiguous cells in the blastoderm [37,39]. In *Xenopus*, this activity has been shown to be enriched in anterior ectoderm at late blastula stages [40]. Dysregulation of this calcium activity results in misalignment of microtubules, which leads ultimately to gastrulation failure [41]. These stages are very challenging to image in mammalian development, so it remains unknown if similar mechanisms are at play in human embryogenesis.

### 2.3. Gastrulation

During gastrulation, embryos exhibit characteristic calcium activity patterns which regulate well-coordinated cell movements that result in the formation of the characteristic vertebrate body plan and the induction of the nervous system. The three germ layers began to form during blastula stages, but become well established and exhibit regional differences during gastrulation [42]. The massive degree of cell movement leads to significant tissue rearrangements and inductive interactions that will ultimately give rise to specific organ systems [43,44]. The ectoderm gives rise to the nervous system and epidermis, while the mesoderm predominantly gives rise to the circulatory system, skeletal-muscular system, connective tissue and urinary system. The digestive, respiratory and glandular systems including organs such as the liver and the pancreas are derived from the endoderm. Typically, failure of gastrulation is embryonic lethal; however, some rare birth defects such as caudal dysgenesis and conjoined twinning are considered to be associated with impaired gastrulation [45].

Not surprisingly, calcium activity is essential for virtually every aspect of gastrulation. Because of the size and accessibility of the embryos, the majority of our knowledge of the critical role of calcium derives from studying amphibian and fish embryos. As embryos progress from the mid-blastula transition to early gastrula stage of development, calcium activity drastically decreases in its frequency of occurrence and its amplitude in zebrafish and *Xenopus* [37,39]. This period of decreased activity, in *Xenopus* and zebrafish embryos, is referred to as a “quiet period” [37,40,46,47]. After mid-blastula transition, however, calcium activity is more complex and tissue specific, that is, different tissue domains of the embryo exhibit differential propagating multicellular waves and non-propagating spikes [39]. For example, the dorsal quadrant of embryos, in comparison with ventral and lateral quadrants, exhibits higher calcium activity in terms of both frequency of occurrence and amplitude in *Xenopus* and zebrafish [37,40,48]. Similarly, calcium activity is enriched in the frequency of occurrence and amplitude in Blastula Chordin and Noggin-expressing region which is located in dorsal animal cells and presumptive neural tissue in *Xenopus* and Zebrafish [40,47,49,50,51]. Additionally, a gradual elevation of Ca^2+^ in the dorsal quadrant is superimposed at early gastrula stages and continues until late neurula in zebrafish and *Xenopus* [40,52]. Immunohistochemical assays have shown that newt embryos express various calcium channels, including L-type voltage-gated calcium channels and ryanodine receptors, throughout the presumptive ectoderm during gastrulation. Inhibition of this calcium activity, using low concentrations of potent inhibitors of the sarco/endoplasmic reticulum Ca^2+^ ATPase pumps, including thapsigargin and cyclopiazonic acid, during late blastula/early gastrula stages of development resulted in severe developmental defects including cyclopia and tail defects in zebrafish [53].

Calcium activity has been shown to regulate the extensive tissue movements that occur during gastrulation; disruption of calcium activity severely impedes cell and tissue movements. During massive tissue rearrangement processes, intercellular calcium waves are generated, which are considered an extension of the spikes and waves observed in the blastula stage [54]. The leading cells of this rearrangement initiate gastrulation by sensing and translating the signal from the ectoderm. Interestingly, these leader cells exhibit remarkably high calcium activity compared to other cells from second and third rows throughout gastrulation in *Xenopus* explants [44,55]. Disruption of calcium activity during gastrulation using calcium chelators such as BAPTA-AM, calcium channel blockers such as R(+)BayK and nicardipine, and morpholino antisense constructs which inhibit translation of calcium channels such as L-type calcium channels and purinergic receptor P2Y11, significantly reduces leader cells’ protrusion activity and migration speed, thus impairing gastrulation and convergent extension [43,44]. When calcium activity was increased by ionomycin treatment, higher migratory activity of the leading edge mesoderm was induced during gastrulation in *Xenopus* [44]. In humans, some extremely rare congenital defects such as sirenomelia and caudal dysgenesis are thought to occur due to impaired gastrulation, however, the relationship to calcium activity remains unclear. In general, defects in gastrulation will result in embryonic lethality [45,56].

### 2.4. Neural Induction

The tissue rearrangements that occur during gastrulation form the basis for neural induction, as the overlying dorsal ectoderm comes into contact with the dorsal mesendoderm. This process commences in the dorsal ectoderm near the onset of gastrulation, from embryonic day 6.0–8.5 in mice, due to a complex interaction between the blastula-chordin and noggin-expressing center and Nieuwkoop center in *Xenopus* [42,57]. This interaction triggers the secretion of neural inducing factors, such as noggin, chordin, follistatin, Xnr3 and Cerberus. These factors inhibit a potent epidermal inducer, bone morphogenetic protein (BMP), and also coordinate regulation of fibroblast growth factor (FGF), Ca^2+^ and Wnt signaling. Therefore, neural induction has been considered the default pathway during development as it occurs in the absence of the suppressive influence of BMP [40,51]. Calcium activity is strongly implicated in the process of neural induction.

Barth and Barth, using amphibian embryos, first introduced the concept that Ca^2+^ treatment can activate neural induction [58]. Stableford (1967) supported this idea by showing an eight-fold increase in Ca^2+^ concentration in the internal fluid of amphibian neurula when compared with the blastula [59]. Recently, further detailed analysis of calcium activity has been achieved with both spatial and temporal resolution during early neural development. These studies have shown that the increase in cytoplasmic Ca^2+^ concentration in the dorsal ectoderm is critical in enabling cross-talk between different signaling pathways, including BMP and FGF/Erk, required for neural induction in *Xenopus* and zebrafish [51,60]. Similar results in mice are consistent with the findings in lower vertebrates. For example, pharmacological inhibition of calcineurin, a calcium-calmodulin-dependent serine/threonine phosphatase, using cyclosporin A and FK506 on day 3 to day 8 of gestation in mice results in reduced expression of neuroectodermal markers *FezF1*, *FezF2*, and *Six3*. This inhibition also increased expression of mesodermal marker Brachyury and BMP responsive genes including BMP4-Smad1/5 target genes, and induced phosphorylation of Smad1/5. However, FGF- or ionophore elevated Ca^2+^ dependent activation of calcineurin rescued these effects [51]. Similarly, when elevation of cytoplasmic Ca^2+^ from neuroectoderm is inhibited using L-type voltage gated calcium channel antagonists, such as R(+)BayK8644, and calcium chelators, such as BAPTA, neuralization of the dorsal ectoderm is prevented. This inhibition blocks the expression of neural markers, including geminin, *Zic3*, and *NCAM* and also results in developmental defects, including head and CNS deformation, absence of eyes, abnormal notochord formation, and spina bifida in *Xenopus* [40]. Similar results have been obtained by inhibiting calcium influx from both the extracellular matrix via other channels including TRP and intracellular stores via mediators such as IP3R and RyR [40,49,51,61]. Taken together, these experiments indicate that calcium signaling is essential for neural induction in a wide range of vertebrate animals, including mammals, and that deregulated calcium activity may result in severe developmental defects.

### 2.5. Organogenesis

Gastrulation establishes the basic vertebrate body plan with recognizable anterior-posterior, dorsal-ventral, and left-right axes. During organogenesis, the regional differences in the three germ layers that developed during the blastula and gastrula stages give rise to different organ systems. While calcium activity is critical for the development of all organ systems, in this review, we will focus on the importance of calcium activity during nervous system, heart, kidney, muscle and immune system development, as well as diseases associated with abnormal calcium activity.

#### 2.5.1. Nervous System Development

##### Neural Tube Closure

Following gastrulation and neural induction, the neural tube forms the rudimentary basis of the central nervous system. By the end of gastrulation, undifferentiated neural progenitor cells form the neural plate on the dorsal surface of the embryo as a flat neuro-epithelial sheet. As embryonic development progresses, this plate lengthens along the anterior-posterior axis in a process called rostro-caudal extension, the lateral borders narrow via a process called mediolateral convergence, elevate, and bend to form ridged/grooved neural folds. The edges of the neural fold meet and fuse to form the hollow neuroepithelial tube. This process is characterized by apical constriction, cell shape change, and morphogenetic movements. Apical constriction is believed to drive convergent extension, movement, and transformation of two-dimensional planar neural tissue to a three-dimensional structure with dorsoventral, mediolateral and rostrocaudal axes in the neural tube between weeks three and four of human gestation [62,63]. This seemingly simple process of neural tube formation is orchestrated by a complex gene expression network, which is tightly regulated by spatiotemporal interactions between different tissue types as well as a number of molecular processes involving ~300 genes, epigenetic modifications, and multiple signaling pathways [63,64].

Calcium activity has been shown to regulate biomechanical processes and epithelial re-modeling during neural tube closure. Smedley and Stanisstreet (1986) cultured rat embryos at 10.4 days in papaverine and D-600, which are pharmacological inhibitors of calcium channels, and found that this inhibition results in severe neural tube defects [65]. This experiment provided the first evidence that calcium is necessary for neural tube closure. Recent studies using vertebrate model organisms including zebrafish, *Xenopus*, and mice have supported the idea that calcium is a major regulator of neural tube closure [4,66,67,68]. Visualization of calcium influxes during neural tube closure using genetically encoded calcium indicator showed distinct spikes and waves. Spikes were short, only lasting less than 40 s, and limited to single cells, while waves were long-lasting transients that originate from a single cell or a group of a few cells and propagate across several to hundreds of cells in *Xenopus* [4,66]. As neural tube closure progresses, intracellular calcium activity increases in terms of both incidence and frequency of occurrence, becoming extremely frequent during late stages of closure. Interestingly, the frequency of calcium activity correlates with a number of biomechanical processes that are necessary for neural tube closure: apical contraction, cell polarization and intercalation, morphogenetic movement, and cell shape change [4,66,68]. The apical regions of neuroepithelial cells are highly enriched with motor proteins that mediate contractions of actin filaments in the apical regions of these cells. When this actin activity is imaged using F-actin marker Lifeact-EGFP at the same time as calcium activity, F-actin mesh-like structures develop in the center of the cells following the calcium transients during neural tube closure in *Xenopus* [4]. In addition, calcium transients trigger non-muscle myosin II activation within a minute of their occurrence in *Xenopus* and *Drosophila* [4,64,67].

Pharmacologically disrupting calcium transients by blocking IP3R and other membrane-localized Ca^2+^ channels, using 2ABP and nifedipine, abolished both apical constriction and movements of the neural plate towards closing. Also, this disruption reduced the width of the neural gene *Sox2* expression zone and ultimately resulted in neural tube closure defects; these defects included impaired forebrain, hindbrain and midbrain in *Xenopus* and *Ciona* [4,66,68,69,70,71]. These results suggest that dysregulation of calcium during convergent extension and neural tube closure results in severe central nervous system defects. Central nervous system defects, including neural tube closure defects, accounted for about 8% of all birth defects in the United States from 2004–2006; examples include anencephaly, spina bifida without anencephaly, encephalocele, and anophthalmia/microphthalmia [72]. The causes of these defects are not completely understood; however, impaired calcium activity is likely to be a potential factor.

##### Neurotransmitter Phenotype Specification

A functional brain is composed of heterogeneous populations of cells that express different neurotransmitters and neurotransmitter receptors. The process of neural differentiation and acquisition of functional identity, also known as neurotransmitter phenotype specification, is achieved by complex interactions between transcription factors, morphogenetic proteins such as BMP and Shh, and other signaling molecules [73]. Common examples of neurotransmitter phenotypes include cholinergic and GABAergic. Interestingly, the neurotransmitter phenotype for a single neuron is plastic and can change depending upon its extrinsic and intrinsic signaling environment [74].

Calcium signaling has been established as a key regulator of neural differentiation and phenotype specification. In 1993, Spitzer and colleagues first introduced the concept of calcium activity-mediated neurotransmitter specification. When presumptive spinal cord neurons of *Xenopus* were cultured in calcium free medium, the total number of GABA immunoreactive GABAergic neurons decreased by 30% [75]. Similarly, when calcium activity of primary neural cells isolated from 3 different stages of development, namely neural plate, neural fold and neural tube, in *Xenopus* was enhanced via exposure to (−)BayK 8644, a pharmacological activator of voltage gated calcium channels, the total number of excitatory neurons was found to be decreased [76]. Furthermore, these results were strengthened by evidence that enhancing calcium spike frequency, either by overexpression of the voltage-gated rat brain sodium channels rNa_v_2aα and rNav_2_aβ or application of the sodium channel antagonist veratridine, resulted in increased GABA and Glycine immunoreactive inhibitory neurons in *Xenopus* embryonic spinal cord in vitro as well as in vivo. Conversely, chronic induction of hyperpolarization, either by overexpression of mRNA encoding the human inward rectifier potassium channel hKir2.1 or pharmacological blockers, including EGTA or a mixture of GVIA w-conotoxin, calcicludine, flunarizine and tetrodotoxin, has been shown to suppress calcium activity. This suppression of calcium activity increased the number of glutamatergic and cholinergic immunoreactive excitatory neurons in *Xenopus* spinal cord, in vivo as well as in tissue culture [77,78]. These changes in the ratio between excitatory neurons and inhibitory neurons lead to neurodevelopmental diseases, including autism spectrum disorder, Rett syndrome, and fragile X syndrome [79,80]. For example, reduction in the number of inhibitory synapses and over-activation of class I metabotropic glutamate receptor in the cerebral cortex and hippocampus due to lack of a modulator of voltage gated calcium channel Cav2.2 called Fragile X Mental Retardation 1(*fmr1*) gene results in fragile X syndrome in mice. Strikingly, when these mice were treated with the GABA agonist gaboxadol, the phenotype was rescued [80].

Furthermore, different cell types have been shown to exhibit their signature frequencies and incidence patterns of calcium activity during a 10-h period of *Xenopus* spinal cord development after neural tube closure. Dorsal sensory Rohon–Beard neurons exhibit spontaneous calcium activity characterized by a low and constant frequency, dorsolateral interneurons exhibit monotonically increasing frequency, ventral motoneurons exhibit low to high stepping frequency, and ventral interneurons maintain a high frequency throughout the larval period [77]. Differential proportions of these different cell types have been implicated in developmental diseases: for example, decreased GABAergic, glutamate decarboxylase 65 KDa isoform immunoreactive, neurons in the cerebellar dentate nuclei and increased GABAergic interneurons in the hippocampus have been implicated in idiopathic autism. Similarly, in vivo studies have associated reduced GABA_A_ receptor density in the fronto-temporal cortex in Rett Syndrome [81]. Involvement of calcium channels in a wide array of disorders associated with impaired neurotransmitter phenotype specification suggests that dysregulation of calcium homeostasis might play a role in the etiology of various pathophysiological conditions, including chronic pain, cerebellar ataxia, autism schizophrenia and migraine [82]. Therefore, dysregulation of calcium signaling could be a potential cause of neurodevelopmental diseases.

#### 2.5.2. Muscle Development

Muscle tissue, both skeletal and cardiac, is the derivative of mesoderm, the middle of the three germ layers. Paraxial mesoderm gives rise to subcutaneous tissue and to somitomeres, which develop into head mesenchyme, skeletal muscles and the axial skeleton, intermediate mesoderm gives rise to urogenital structures, and lateral plate mesoderm gives rise to components of limbs and the circulatory system, including the heart. It is apparent that derivation of these structures from a primitive germ layer is the product of a complex spatio-temporally regulated interaction network which is affected by multiple factors, calcium being one of them. Disruption of calcium activity during muscle development results in severe developmental defects, including but not limited to reduced and amorphous skeletal muscle [83,84]. Differentiating muscle cells, both in culture and in situ, exhibit spontaneous calcium transients as a first step of muscle development (myogenesis) in vertebrates. Chernoff and Hilfer for the first time cultured chick embryo trunk in media supplemented with and without calcium, in addition to various other calcium transport agonists and antagonists, including caffeine, ionophore A23187, papaverine, and verapamil, and determined that calcium activity was required for somitogenesis [85]. In addition, somite maturation and calcium activity have been shown to correlate in *Xenopus*. When somites of early tailbud stage *Xenopus* embryos, namely anterior somites, maturing somites, segmenting somites and unsegmented paraxial mesoderm, were exposed and imaged using a calcium sensitive dye (fluo-3) along the AP-axis, calcium activity, measured by incidence, frequency and duration of transients, was inversely correlated with the maturity of somites. For instance, anterior somites were more mature than unsegmented paraxial mesoderm as reflected in the expression of sarcomeric myosin, and anterior somites exhibited no calcium activity while unsegmented paraxial mesoderm exhibited the highest activity [86]. Interestingly, two distinct calcium active periods with a frequency ranging from 0.02 Hz to 0.12 Hz and a quiet period lasting about 3.5 h at the 28-somite stage have been shown to occur during slow muscle cell development after the 17-somite stage in transgenic zebrafish. This transgenic zebrafish constitutively expressed apoaequorin under an actin promoter only in developing muscle cells [87,88,89]. All of these observations underscore the importance of calcium during early muscle development.

Calcium activity observed during myogenesis, as opposed to neurogenesis, mainly relies upon store-operated calcium entry, which is mediated by RyR, TPC2, IP3R, ORAI/STIM, VGCC, and TRPC, among others [90,91,92]. Dysregulation of cytoplasmic Ca^2+^ homeostasis due to mutations or pharmacological manipulation of these mediators during myogenesis results in severe fetal neuromuscular disorders [82,93,94]. For instance, mice lacking STIM1 die perinatally [95]. Loss of function mutations in *STIM1* and *Orai* have also been identified in human patients suffering from hypotonia [96,97]. Additionally, blocking the the RyR channel using ryanodine resulted in inhibited differentiation of fetal myoblasts in E9.5 mice [98], hydrocephaly and scoliokyphosis in *Xenopus* [86]; and disruption of the vertical myoseptae and smooth muscle cell spanning in zebrafish [87,99]. Also, a homozygous Ile4898 to I4898T mutation in RYR1 in E9.5 mice causes severe asphyxia characterized by reduced and amorphous skeletal muscle, disorganized myofibrils, delayed cardiovascular development and perinatal death [92]. Similarly, when TPC2 expression was blocked using a TPC2 specific morpholino or a CRISPR/Cas system, decreased myotome width, U-shaped somites in the anterior trunk instead of the usual chevron-shaped, and smooth muscle cell myofibrils that were not aligned into bundles were observed in zebrafish [88,89]. These phenotypic effects were rescued when additional calcium release from intracellular stores was triggered using caffeine, IP3 or expression of mRNA construct of TPC2 [88,89]. These observations demonstrate the crucial role of calcium signaling in diseases of the muscle.

#### 2.5.3. Heart Development

The heart and other components of the circulatory system derive from lateral plate mesoderm during the third week of pregnancy in humans. A plate of promyocardial cells intermixed with endothelial strands on either side of the neural fold represents the developing heart at the neural fold stage of development. As embryos progress, two massive growth events, a massive growth of the anterior portion of the neural tube and an endodermal invagination to form the foregut, drive the folding of the promyocardial plate to form endocardial tubes in either side of the neural tube. At this stage, the heart is bilaterally symmetrical, with an inverted Y shape. Although establishment of the pulmonary system happens later in development, the endothelial strand ensures the presence of the circulatory system. These two tubes elongate and converge towards each other to form the primitive heart tube, which quickly forms different cardiac structures including the truncus arteriosus and sinus venosus [100,101,102]. It is indisputable that a genetic blueprint is required to regulate all the developmental processes and necessary cell differentiations in the developing heart, including formation of cardiomyocytes and valvular interstitial cells. For example, NKX2.5 is expressed in early heart progenitor cells, and is considered a master controller of cardiac development. In addition, various members of the TGF-β superfamily including nodal and activin, BMPs, and other signaling molecules including calcium have been shown to play an indispensable role during cardiogenesis [100].

Dysregulation of calcium activity results in failure in every step of cardiac development, including differentiation of cardiac progenitor cells and cardiac tube formation. Two decades ago, using aequorin, Creton et al. found a 10-fold increase in cytoplasmic Ca^2+^ in the cardiac region during cardiogenesis at the segmentation period of zebrafish development. This increase was achieved by a large number of calcium spikes that occurred every 10–20 min for several hours [103]. When this calcium activity was blocked by injecting low concentrations of BAPTA buffer into the zygote (higher concentrations stalled fertilized embryos in the one cell stage), the heart defects were apparent. The heart was smaller and stretched longitudinally, and it was not able to pump the blood [103]. Recent findings using a diverse group of organisms, both in vivo and in vitro, have strengthened our understanding of calcium’s role in cardiac development. For example, elevated levels of calcium were observed while imaging the heart region in both transgenic zebrafish and mice at comparable stages of development. These transgenic animals expressed genetically encoded calcium markers GCaMP6 and GCaMP2 respectively, which express under the promoter of ubiquitously expressing genes (Tg[βactin2:GCaMP6s]stl351, Tg[ubi:GCaMP6s]stl352 in zebrafish and Tet-Off αMHC-CaMP2 in mice) in order to visualize cytosolic Ca^2+^ ions [37,104]. When these calcium spikes during cardiogenesis are blocked by culturing mouse embryos at E7.5 to E8.5 in media supplemented with the L-type voltage gated calcium channel blockers, nifedipine and verapamil, various heart defects, including lack of a right ventricle and a large left ventricle, were apparent [105]. In addition, these blockers reduced DNA synthesis, (assayed using tritiated [3H]-thymidine incorporation), cell division (via reduction of the mRNA expression of a positive cell cycle regulator), cyclin B1 (detected using qPCR), and differentiation of cardiac progenitors (detected using immunolabeling of differentiation marker sarcomeric myosin), in cardiac cells from E11.5 mice in vitro [106]. Similarly, mouse embryos treated with bivalent cation chelators such as BAPTA or EGTA, or embryos that lack calreticulin, which is a Ca^2+^-binding chaperone of the endoplasmic reticulum, die in utero due to defective heart development. However, the calcium ionophore ionomycin restored myofibrillogenesis in cardiomyocytes [107,108]. These observations underscore the importance of calcium activity during cardiogenesis and associated developmental heart defects.

#### 2.5.4. Kidney Development

Disruptions to calcium signaling pathways have also been implicated in a number of developmental dysfunctions and diseases in the kidney. Calcium activity mediates the initiation and formation of the kidney field; the kidney field is defined as the mesodermal territory where expression of *pax8* and *lhx1* overlap during organogenesis. Overexpression of *pax8* and/or *lhx1* leads to the formation of enlarged and ectopic pronephroi [52,109]. In addition, calcium regulates the transcription of genes that are involved in the formation of renal structures, including *pax8*, *lhx1*, *osr1*, and *osr2*. Loss of function of any of these genes leads to impaired pronephros development in *Drosophila* and *Xenopus* [109,110,111]. Using aequorin imaging, it was shown that calcium transients occur in intact *Xenopus* embryos in the lateral mesoderm during kidney tubule formation. When these transients were inhibited with a calcium chelator during the late gastrula or mid neurula stage, kidney tubule development was disrupted. Interestingly, incubating *Xenopus* ectoderm with activin A, which is a IP3 modulator/mesodermal inducer, followed by retinoic acid, which is a voltage-gated calcium channels modulator, rescued pronephric tubule formation [112]. Note that retinoic acid stimulates calcium transients, while activin A alone does not. However, treating *Xenopus* ectoderm with activin A followed by caffeine or ionomycin not only increased cytoplasmic Ca^2+^, but also triggered normal tubule differentiation [112]. This evidence suggests that calcium signaling is necessary and sufficient to regulate the tubule differentiation process.

Additionally, several calcium-dependent proteins have been linked to pronephros development and function. For example, the proteins polycystin-1 and polycystin-2, encoded by the genes PKD1 and PKD2 respectively, are expressed in renal tissue of both humans and mice during renal development and have been shown to function together to create a calcium-permeable channel [107]. Mutations in PKD1 and PKD2 result in autosomal dominant polycystic kidney disease in humans [113]. The protein products of these mutant forms of PKD1 and PKD2 do not form their normal calcium permeable channel during embryogenesis, which results in the development of large fluid-filled cysts within the kidney tubules and collecting ducts. Collectively these observations suggest that calcium activity is instrumental to multiple aspects of kidney development and associated diseases.

#### 2.5.5. Immune System

Although the immune system does not appear until later stages of development, for example until 12 weeks of development in humans, immune cell differentiation and migration, which are mediated by calcium signaling, are hallmarks of development. Boucek and Snyderman first demonstrated the requirement for calcium activity during neutrophil functioning using lanthanum chloride, which is a calcium influx inhibitor [114]. More recent studies have strengthened the idea that a transient increase in cytoplasmic Ca^2+^ is required for immune cell differentiation and migration. *Stim1* and *Stim2* double-knockout mice at 5–6 weeks showed reduced regulatory T cells in the thymus, spleen, and lymph nodes, and these T-cells showed no Ca^2+^ influx when imaged using calcium indicator Fura-2-AM even after thapsigargin treatment [115]. These knockout mice exhibited a decrease in regulatory T-cells in all immune organs, leading to autoimmune disease symptoms [115,116]. Similarly, imaging transgenic zebrafish embryos that expressed GCaMP3 specifically in neutrophils under the promoter *LysC* showed elevated cytoplasmic Ca^2+^ as well as enhanced calcium spiking behavior during migration and phagocytosis at wound sites in vivo. Inhibition of this calcium activity using the calcium channel antagonist SKF 96365 resulted in impaired recruitment of these neutrophils due to their undirected movement [117]. Likewise, when fura-2 loaded human neutrophils were cultured in media with and without calcium supplement, cells cultured in calcium free medium were unable to migrate on poly-d-lysine-coated glass [118].

In addition, cytoplasmic Ca^2+^ elevation is also facilitated by store-operated calcium entry through calcium release activated channels in immune cells, including lymphocytes and mast cells [115,119]. Dysregulation of calcium release activated channels results in a severely compromised immune system. For example, mice lacking STIM1 or with a gene-trap insertion in the *Orai1* gene show defective mast cell degranulation and hereditary immunodeficiencies [120,121]. Patients with these mutations displayed symptoms such as severe T cell immunodeficiency, impaired T cell activation and proliferation leading to recurrent viral, bacterial, and fungal infections, and muscular hypotonia [119]. Similarly, alterations in *STIM1* expression affected the sensitivity of immunoglobin E-mediated immediate-phase anaphylactic responses in vivo in mice [120]. These observations underscore the importance of calcium homeostasis and signaling behaviors in immune system development and function.

## 3. Wound Healing and Regeneration

### 3.1. Wound Healing before Formation of Immune System

In order to reach maturity, embryos must be able to respond to a wide variety of perturbations that are likely to occur during development. Embryos in the early stages of development exhibit inflammatory-response-free and scar-free wound closure with a mechanism that closely parallels neural tube closure during morphogenesis [122]. This healing is often called regenerative healing, and does not occur in most adult organisms [123]. The mechanisms of these abilities rely mostly on calcium signaling. Imaging the optic tectum of *Xenopus* larva using Oregon Green BAPTA-1 showed that fine micropipette mediated mechanical insults and targeted induction of cell death by a high-voltage electrical stimulus triggered a rapid influx of Ca^2+^ that generated further spontaneous Ca^2+^ influxes, which propagated through multiple rows of cells surrounding the injury site within seconds. These rapid calcium waves were accompanied by contractions of the neuroepithelium and expulsion of potentially damaged cells [124]. When this calcium activity was blocked and/or inhibited using BAPTA, thapsigargin, 2-aminoethoxydiphenyl borate, which is an IP3R blocker, and an array of purinergic receptor blockers, tissue contractions and potentially damaged cell expulsions were hindered. This blockage also negatively impacted wound healing [124]. In addition, damaged cells also release ATP into the extracellular milieu, which is recognized by the metabotropic purinergic receptors P2Ys on adjacent undamaged cells [125]. Activation of P2Y receptors further increases the cytoplasmic Ca^2+^ and activated metalloproteinase. This leads to heparin-binding EGF-like growth factor activation and subsequent cell proliferation, as well as formation of actomyosin cables and actin-rich protrusions, which guide tissue contraction, cell elongation, and migration in order to close the wound [125]. Therefore, calcium activity plays an indispensable role in embryonic scar-free regenerative wound healing.

### 3.2. Wound Healing Following the Formation of Immune System

At later stages of development, following the formation of functional immune cells, wound healing differs mechanistically from early embryonic wound healing, however, calcium activity continues to play a critical role. During this later stage of development, immune cells are also involved in wound healing and healing is no longer inflammation-response-free. Interestingly, this mechanism of healing is also regulated by calcium as a first responder. In this mechanism, calcium regulates both modulation of actomyosin and recruitment of immune cells. In *Drosophila* embryos, elevated calcium induced by laser wounding was shown to activate hydrogen peroxide synthase, resulting in a rapid accumulation of H_2_O_2_ around wound sites as indicated by the fluorigenic reporter Amplex Ultrared. A reduction in H_2_O_2_ signals at the wound site was observed in TRPM2 channel and innexin 2 mutant embryos, in which calcium elevation did not occur, indicating that calcium was necessary to this inflammatory response [126]. Likewise, using a genetically encoded H_2_O_2_ ratiometric sensor HyPer, Niethammer et al. for the first time showed that H_2_O_2_ is required for rapid recruitment of leukocytes to the wound in zebrafish larva [127]. Inhibition of this calcium activity using thapsigargin or EGTA not only inhibits H_2_O_2_ release healing efficiency, but also reduces the average hemocyte response during laser-induced epithelial wound healing in *Drosophila* embryos [126]. Even after the immune system develops and matures, calcium continues to play a critical role in embryonic wound healing.

### 3.3. Regeneration

Equally importantly, embryos also show calcium-mediated tissue regenerative capabilities during the early stages of development. Spontaneous calcium activity has been observed in the regenerating tail in *Xenopus* during the first hours of recovery. Inhibiting this calcium activity using ryanodine reduced the number of activated muscle progenitor cells and skeletal muscle stem cells, also known as muscle satellite cells, in the regenerating tissue. This, in turn, inhibited the regeneration process [128]. During regeneration, calcium activity increases secretion and mobilization of growth factors including insulin-like growth factor, interleukins, including interleukin-1, interleukin-6 and interleukin-8, parathyroid hormone, transforming growth factor-β and platelet-derived growth factor. Platelets are widely recognized as inducers of cell proliferation, and are also involved in stem cell differentiation, cell migration, and revascularization of damaged tissue via increased release of growth factors during tissue regeneration. Therefore, platelet-rich plasma derivatives have been used in regenerative medicine [129]. This increase in growth factor release has been proven- to be mediated by calcium activity. This calcium activity has been shown to be critical for platelet functioning using double knockouts of various calcium channels, including TRPM7, TRPC6, Orai1 and SERCA, and pharmacological agonists and antagonists in mice [130,131,132]. These factors facilitate changes in the expression of specific genes that are responsible for proper cell proliferation and migration during regeneration [122,133]. Using microarrays, Patterson et al. reported 624 upregulated and 826 downregulated genes associated with epidermal regeneration in *Drosophila* embryos, including genes related to cellular component organization and stress response [134]. Similarly, using an RNA-Seq approach, Tsujioka et al. reported 25 candidate genes involved in tail regeneration in *Xenopus*, including genes related to cell proliferation, for example, CDK1 and cyclin B2 [135]. These results indicate that calcium activates expression of a number of gene cascades that are responsible for tissue regeneration. Understanding the underlying mechanisms of embryonic regeneration could lead to new developments in the fields of regenerative clinical medicine and tissue engineering.

## 4. Conclusions and Future Directions

**Figure 1:**
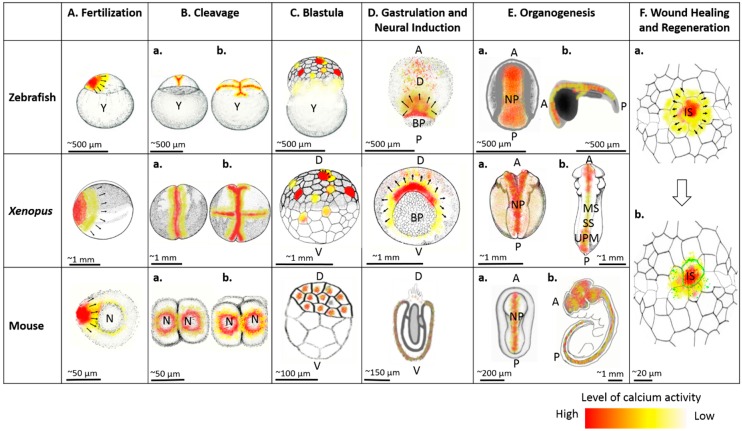
Comparative calcium activity among zebrafish, frogs, and mice during egg activation and fertilization (**A**), first cleavage (**Ba**), second cleavage (**Bb**), blastula (**C**), gastrulation and neural induction (**D**) and organogenesis (**E**), including neural tube closure (**Ea**) and muscle development (**Eb**) in zebrafish, *Xenopus*, and mouse; also, depiction of calcium dynamics immediately after tissue damage (**Fa**) and calcium mediated actomyosin filament (green lining) during wound healing and regeneration (**Fb**). Black arrows show direction of propagation of calcium waves. Y, yolk; A, anterior; P, posterior; D, dorsal; V, ventral; BP, blastopore; NP, neural plate; UPM, unsegmented paraxial mesoderm; MS, matured somites; SS, segmenting somites; IS, injury site; N, nucleus. Images were adapted and re-drawn from [136,137,138].

Over the last three decades, studies have shown that dysregulation of calcium signaling results in severe medical conditions, including neural tube and other developmental defects. Given the versatility of calcium as a signaling molecule and its near ubiquitous involvement with virtually every aspect of embryonic development (Figure 1), this is scarcely surprising. Yet despite its importance in development and the genesis of human disease, there is much that we do not understand about calcium and many avenues for future research. For example, currently, a comprehensive analysis of the regulation of spatiotemporal expression patterns of calcium-associated genes and their interactions is lacking. Such an analysis would suggest specific tissues and developmental timepoints where calcium-regulated processes can go awry. New gene editing and tissue engineering techniques as well as novel next generation sequencing approaches may aid in understanding the etiology and mechanisms of medical conditions associated with dysregulation of calcium, as well as potential therapeutic solutions for these conditions. In addition, determination of the embryonic expression patterns, both at the mRNA and protein level, of genes known to be involved in calcium activity and screens for novel genes implicated in calcium activity are warranted.

In addition to many avenues for further research, there is also a huge potential to utilize this versatile signaling molecule therapeutically and in regenerative medicine. It is well known that developing embryos up to the blastocyst stage of development and embryonic stem cells exhibit the ability to either maintain stem cell identity or differentiate into the lineages of all three germ layers. This opens new avenues for the exploration of these cells’ potential uses in regenerative medicine [139,140,141]. The ultimate goal of regenerative medicine is to restore or substitute damaged tissue so the tissue can maintain its function(s); this may require boosting natural defense systems, which could harness and accelerate innate healing processes in order to cure previously unmanageable medical problems [142]. Towards this end, Jergova et al. engineered neural progenitor cells to generate transgenic GABAergic cells that release an analgesic peptide serine-histogranin, which antagonizes NMDA receptors. These cells were then transplanted onto an injury site in order to assess their effect on peripheral neuropathic pain after 1 week of induced injury in rat spinal cord. This transplant resulted in improved pain sensitivity based upon a pain-related behavioral assessment with four pain parameters: mechanical, heat, cold, and tactile [143]. Similarly, Mery et al. engineered pacemaker cells from embryonic stem cells that induce InsP3 mediated oscillatory calcium release from the endoplasmic reticulum; these cells had a potential to serve as pacemaker cells prior to the development of pacemaker ionic channels in early embryos. This oscillatory calcium release was required for pacemaker activity. When InsP3 receptor antisense cDNA was expressed constitutively in these cells, expression of type I InsP3 receptors and spontaneous calcium activity, both frequency of occurrence and amplitude, were reduced, and beating activity was also impaired [144]. These observations underscore the potential use of calcium signaling toolkits in medical cell and tissue engineering.

Current approaches used to understand calcium-related defects include mapping and cloning causative gene(s), recapitulating clinical features, and using pharmacological reagents in order to ameliorate symptoms. Gene editing is also beginning to be explored as a way to understand and possibly treat these defects. For example, Limpitikul et al. reprogrammed dermal fibroblasts into iPSC-derived cardiomyocytes, then they used these cardiomyocytes to model calm2-associated Long-QT syndrome, which is a congenital birth defect associated with a mutation in the calcium binding protein calmodulin2, *calm2* gene. Using CRISPRi technology, they were able to selectively correct the mutated allele and rescue the long QT syndrome phenotype [145]. Molecular gene editing tools like CRISPR have tremendous capabilities for therapeutic use, and these capabilities will continue to expand as molecular technology improves even further. However, off-target effects of these tools and complex etiologies of some birth defects continue to create critical therapeutic challenges. These etiologies can be influenced by a wide array of factors, including multiple genes, impaired gene-gene interactions, combinatorial effects of polymorphisms, changes in protein levels in time and space, epigenetic modifications, environmental effects, and mutations in epigenetic regulators. For example, ~300 genes have been shown to be critically required for neural tube closure [63], and RNAseq data identified at least 30 causative genes for congenital fetal akinesia deformation [83]. Addressing all of these factors during the process of therapeutic development remains a challenge in the scientific community.

Taken together, these results indicate that calcium is involved in a diverse array of physiological processes, and dysregulation of calcium activity results in wide array of developmental defects. There is a tremendous demand for scar-free healing after major surgical procedures, an ability the embryo possesses following unique patterns of calcium activity. The capacity of the embryo to either maintain stem cell identity or differentiate into specific lineages such as neural cells could also be used to treat neurodegenerative diseases. While great progress has been made in recent decades, translation of these fetal abilities into adult systems still remains a challenge. Understanding how an ion as simple as Ca^2+^ plays diverse physiological roles may help solve these medical problems. Some aspects of calcium signaling are understood in a variety of processes already, and recent advances in genomics, transcriptomics, proteomics, and gene editing technology may help expand our knowledge about the underlying mechanisms of calcium function and enable its potential therapeutic use.

## Supplementary Materials

Supplementary materials can be found at http://www.mdpi.com/1422-0067/19/11/3390/s1.

## References

[1] Database GeneCards GeneCards—Human Genes|Gene Database|Gene Search. https://www.genecards.org/.

[2] Lew V.L., Tsien R.Y., Miner C., Bookchin R.M. (1982). Physiological [Ca^2+^]Ilevel and Pump-Leak Turnover in Intact Red Cells Measured Using an Incorporated Ca Chelator. Nature.

[3] Kramer I. (2016). Intracellular Calcium. Signal Transduction.

[4] Suzuki M., Sato M., Koyama H., Hara Y., Hayashi K., Yasue N., Imamura H., Fujimori T., Nagai T., Campbell R.E. (2017). Distinct Intracellular Ca^2+^ Dynamics Regulate Apical Constriction and Differentially Contribute to Neural Tube Closure. Development.

[5] Balaji R., Bielmeier C., Harz H., Bates J., Stadler C., Hildebrand A., Classen A.K. (2017). Calcium Spikes, Waves and Oscillations in a Large, Patterned Epithelial Tissue. Sci. Rep..

[6] Suzuki J., Kanemaru K., Ishii K., Ohkura M., Okubo Y., Iino M. (2014). Imaging Intraorganellar Ca^2+^ at Subcellular Resolution Using CEPIA. Nat. Commun..

[7] Berridge M.J., Lipp P., Bootman M.D. (2000). The Versatility and Universality of Calcium Signalling. Nat. Rev. Mol. Cell Biol..

[8] Dupont G., Combettes L., Leybaert L. (2007). Calcium Dynamics: Spatio-Temporal Organization from the Subcellular to the Organ Level. Int. Rev. Cytol..

[9] Markova O., Sénatore S., Chardès C., Lenne P.F. (2015). Calcium Spikes in Epithelium: Study on Drosophila Early Embryos. Sci. Rep..

[10] Gu X., Olson E.C., Spitzer N.C. (1994). Spontaneous Neuronal Calcium Spikes and Waves during Early Differentiation. J. Neurosci. Off. J. Soc. Neurosci..

[11] Clapham D.E. (2007). Calcium Signaling. Cell.

[12] Berridge M.J., Bootman M.D., Roderick H.L. (2003). Calcium Signalling: Dynamics, Homeostasis and Remodelling. Nat. Rev. Mol. Cell Biol..

[13] National Institutes of Health National Center for Advancing Translational Sciences (NCATS). https://ncats.nih.gov/.

[14] Zhaurova K. (2008). Genetic Causes of Adult-Onset Disorders. Nat. Educ..

[15] Robertson S., Lin R. (2018). Oocyte-to-Zygote Transition. Semin. Cell Dev. Biol..

[16] Kuroda K., Brosens J.J., Quenby S., Takeda S. (2018). Treatment Strategy for Unexplained Infertility and Recurrent Miscarriage.

[17] Kashir J., Deguchi R., Jones C., Coward K., Stricker S.A. (2013). Comparative Biology of Sperm Factors and Fertilization-Induced Calcium Signals across the Animal Kingdom. Mol. Reprod. Dev..

[18] Wozniak K.L., Mayfield B.L., Duray A.M., Tembo M., Beleny D.O., Napolitano M.A., Sauer M.L., Wisner B.W., Carlson A.E. (2017). Extracellular Ca^2+^ Is Required for Fertilization in the African Clawed Frog, Xenopus Laevis. PLoS ONE.

[19] Mizuno H., Sassa T., Higashijima S., Okamoto H., Miyawaki A. (2013). Transgenic Zebrafish for Ratiometric Imaging of Cytosolic and Mitochondrial Ca^2+^ response in Teleost Embryo. Cell Calcium.

[20] Cuthbertson K.S., Whittingham D.G., Cobbold P.H. (1981). Free Ca^2+^ Increases in Exponential Phases during Mouse Oocyte Activation. Nature.

[21] Deguchi R., Shirakawa H., Oda S., Mohri T., Miyazaki S. (2000). Spatiotemporal Analysis of Ca(2+) Waves in Relation to the Sperm Entry Site and Animal-Vegetal Axis during Ca(2+) Oscillations in Fertilized Mouse Eggs. Dev. Biol..

[22] Ferrer-Buitrago M., Bonte D., De Sutter P., Leybaert L., Heindryckx B. (2018). Single Ca^2+^ Transients vs. Oscillatory Ca^2+^ Signaling for Assisted Oocyte Activation: Limitations and Benefits. Reproduction.

[23] Miao Y.-L., Stein P., Jefferson W.N., Padilla-Banks E., Williams C.J. (2012). Calcium Influx-Mediated Signaling Is Required for Complete Mouse Egg Activation. Proc. Natl. Acad. Sci. USA.

[24] Miao Y.L., Williams C.J. (2012). Calcium Signaling in Mammalian Egg Activation and Embryo Development: The Influence of Subcellular Localization. Mol. Reprod. Dev..

[25] Bernhardt M.L., Zhang Y., Erxleben C.F., Padilla-Banks E., McDonough C.E., Miao Y.-L., Armstrong D.L., Williams C.J. (2015). CaV3.2 T-Type Channels Mediate Ca^2+^ Entry during Oocyte Maturation and Following Fertilization. J. Cell Sci..

[26] Tang T.S., Dong J.B., Huang X.Y., Sun F.Z., Royse J., Blayney L.M., Swann K., Lai F.A. (2000). Ca(2+) Oscillations Induced by a Cytosolic Sperm Protein Factor Are Mediated by a Maternal Machinery That Functions only Once in Mammalian Eggs. Development.

[27] Saunders C.M. (2002). Ca^2+^ Oscillations Triggered by Sperm PLCz.

[28] Hachem A., Godwin J., Ruas M., Lee H.C., Ferrer Buitrago M., Ardestani G., Bassett A., Fox S., Navarrete F., de Sutter P. (2017). PLCζ Is the Physiological Trigger of the Ca^2+^ Oscillations That Induce Embryogenesis in Mammals but Conception Can Occur in Its Absence. Development.

[29] Lee K., Wang C., Machaty Z. (2012). STIM1 Is Required for Ca^2+^ Signaling during Mammalian Fertilization. Dev. Biol..

[30] Yamaguchi T., Kuroda K., Tanaka A., Watanabe S. (2018). Fertilization Failure. Treatment Strategy for Unexplained Infertility and Recurrent Miscarriage.

[31] Ferrer-Buitrago M., Dhaenens L., Lu Y., Bonte D., Vanden Meerschaut F., De Sutter P., Leybaert L., Heindryckx B. (2018). Human Oocyte Calcium Analysis Predicts the Response to Assisted Oocyte Activation in Patients Experiencing Fertilization Failure after ICSI. Hum. Reprod..

[32] Halliday J.L., Ukoumunne O.C., Baker H.W.G., Breheny S., Jaques A.M., Garrett C., Healy D., Amor D. (2010). Increased Risk of Blastogenesis Birth Defects, Arising in the First 4 Weeks of Pregnancy, after Assisted Reproductive Technologies. Hum. Reprod..

[33] Chen C., Jin J., Lee G.A., Silva E., Donoghue M. (2016). Cross-Species Functional Analyses Reveal Shared and Separate Roles for Sox11 in Frog Primary Neurogenesis and Mouse Cortical Neuronal Differentiation. Biol. Open.

[34] Chan C.M., Chen Y., Hung T.S., Miller A.L., Shipley A.M., Webb S.E. (2015). Inhibition of SOCE Disrupts Cytokinesis in Zebrafish Embryos via Inhibition of Cleavage Furrow Deepening. Int. J. Dev. Biol..

[35] Chan C.M., Aw J.T.M., Webb S.E., Miller A.L. (2016). SOCE Proteins, STIM1 and Orai1, Are Localized to the Cleavage Furrow during Cytokinesis of the First and Second Cell Division Cycles in Zebrafish Embryos. Zygote.

[36] Eno C., Gomez T., Slusarski D.C., Pelegri F. (2018). Slow Calcium Waves Mediate Furrow Microtubule Reorganization and Germ Plasm Compaction in the Early Zebrafish Embryo. Development.

[37] Chen J., Xia L., Bruchas M.R., Solnica-Krezel L. (2017). Imaging Early Embryonic Calcium Activity with GCaMP6s Transgenic Zebrafish. Dev. Biol..

[38] Webb S.E., Miller A.L. (2006). Ca^2+^ Signaling during Vertebrate Somitogenesis. Acta Pharmacol. Sin..

[39] Ma L.H., Webb S.E., Chan C.M., Zhang J., Miller A.L. (2009). Establishment of a Transitory Dorsal-Biased Window of Localized Ca^2+^ Signaling in the Superficial Epithelium Following the Mid-Blastula Transition in Zebrafish Embryos. Dev. Biol..

[40] Leclerc C., Webb S.E., Daguzan C., Moreau M., Miller A.L. (2000). Imaging Patterns of Calcium Transients during Neural Induction in Xenopus Laevis Embryos. J. Cell Sci..

[41] Hara Y., Nagayama K., Yamamoto T.S., Matsumoto T., Suzuki M., Ueno N. (2013). Directional Migration of Leading-Edge Mesoderm Generates Physical Forces: Implication in Xenopus Notochord Formation during Gastrulation. Dev. Biol..

[42] Levine A.J., Brivanlou A.H. (2007). Proposal of a Model of Mammalian Neural Induction. Dev. Biol..

[43] Shindo A., Hara Y., Yamamoto T.S., Ohkura M., Nakai J., Ueno N. (2010). Tissue-Tissue Interaction-Triggered Calcium Elevation Is Required for Cell Polarization during Xenopus Gastrulation. PLoS ONE.

[44] Hayashi K., Yamamoto T.S., Ueno N. (2018). Intracellular Calcium Signal at the Leading Edge Regulates Mesodermal Sheet Migration during Xenopus Gastrulation. Sci. Rep..

[45] Ferrer-Vaquer A., Hadjantonakis A.K. (2013). Birth Defects Associated with Perturbations in Preimplantation, Gastrulation, and Axis Extension: From Conjoined Twinning to Caudal Dysgenesis. Wiley Interdiscip. Rev. Dev. Biol..

[46] Gilland E., Miller A.L., Karplus E., Baker R., Webb S.E. (1999). Imaging of Multicellular Large-Scale Rhythmic Calcium Waves during Zebrafish Gastrulation. Proc. Natl. Acad. Sci. USA.

[47] Webb S.E., Moreau M., Leclerc C., Miller A.L. (2005). Calcium Transients and Neural Induction in Vertebrates. Cell Calcium.

[48] Yuen M.Y.F., Webb S.E., Chan C.M., Thisse B., Thisse C., Miller A.L. (2013). Characterization of Ca^2+^ signaling in the External Yolk Syncytial Layer during the Late Blastula and Early Gastrula Periods of Zebrafish Development. Biochim. Biophys. Acta.

[49] Moreau M., Neant I., Webb S.E., Miller A.L., Leclerc C. (2008). Calcium Signalling during Neural Induction in Xenopus Laevis Embryos. Philos. Trans. R. Soc. B Biol. Sci..

[50] Leclerc C., Néant I., Moreau M. (2011). Early Neural Development in Vertebrates Is also a Matter of Calcium. Biochimie.

[51] Cho A., Tang Y., Davila J., Deng S., Chen L., Miller E., Wernig M., Graef I.A. (2014). Calcineurin Signaling Regulates Neural Induction through Antagonizing the BMP Pathway. Neuron.

[52] Moreau M., Néant I., Webb S.E., Miller A.L., Riou J.F., Leclerc C. (2016). Ca^2+^ coding and Decoding Strategies for the Specification of Neural and Renal Precursor Cells during Development. Cell Calcium.

[53] Creton R. (2004). The Calcium Pump of the Endoplasmic Reticulum Plays a Role in Midline Signaling during Early Zebrafish Development. Dev. Brain Res..

[54] Webb S.E., Miller A.L. (2006). Ca^2+^ signaling and Early Embryonic Patterning during the Blastula and Gastrula Periods of Zebrafish and Xenopus Development. Biochim. Biophys. Acta.

[55] Wallingford J.B., Ewald A.J., Harland R.M., Fraser S.E. (2001). Calcium Signaling during Convergent Extension in Xenopus. Curr. Biol..

[56] Lange L., Marks M., Liu J., Wittler L., Bauer H., Piehl S., Bläß G., Timmermann B., Herrmann B.G. (2017). Patterning and Gastrulation Defects Caused by the Tw18 Lethal Are Due to Loss of Ppp2r1a. Biol. Open.

[57] Yasuoka Y., Taira M. (2018). The Molecular Basis of the Gastrula Organizer in Amphibians and Cnidarians. Reproductive and Developmental Strategies. Diversity and Commonality in Animals.

[58] Barth L.G., Barth L.J. (1964). Sequential Induction of the Presumptive Epidermis of the Rana Pipiens Gastrula. Biol. Bull..

[59] Stableford L.T. (1967). A Study of Calcium in the Early Development of the Amphibian Embryo. Dev. Biol..

[60] Leclerc C., Néant I., Moreau M. (2012). The Calcium: An Early Signal That Initiates the Formation of the Nervous System during Embryogenesis. Front. Mol. Neurosci..

[61] Lee H.-K., Lee H.-S., Moody S.A. (2014). Neural Transcription Factors: From Embryos to Neural Stem Cells. Mol. Cells.

[62] Flanagan M., Sonnen J.A., Keene C.D., Hevner R.F., Montine T.J. (2018). Molecular Basis of Diseases of the Nervous System.

[63] Wilde J.J., Petersen J.R., Niswander L. (2014). Genetic, Epigenetic, and Environmental Contributions to Neural Tube Closure. Annu. Rev. Genet..

[64] Nikolopoulou E., Galea G.L., Rolo A., Greene N.D.E., Copp A.J. (2017). Neural Tube Closure: Cellular, Molecular and Biomechanical Mechanisms. Development.

[65] Smedley M.J., Stanisstreet M. (1986). Calcium and Neurulation in Mammalian Embryos II. Effects of Cytoskeletal Inhibitors and Calcium Antagonists on the Neural Folds of Rat Embryos. Development.

[66] Christodoulou N., Skourides P.A.A. (2015). Cell-Autonomous Ca^2+^ Flashes Elicit Pulsed Contractions of an Apical Actin Network to Drive Apical Constriction during Neural Tube Closure. Cell Rep..

[67] Kong D., Wolf F., Großhans J. (2018). In Vivo Optochemical Control of Cell Contractility at Single Cell Resolution by Ca^2+^ Induced Myosin Activation. bioRxiv.

[68] Sahu S.U., Visetsouk M.R., Garde R.J., Hennes L., Kwas C., Gutzman J.H. (2017). Calcium Signals Drive Cell Shape Changes during Zebrafish Midbrain–hindbrain Boundary Formation. Mol. Biol. Cell.

[69] Abdul-Wajid S., Morales-Diaz H., Khairallah S.M., Smith W.C. (2015). T-Type Calcium Channel Regulation of Neural Tube Closure and EphrinA/EPHA Expression. Cell Rep..

[70] Sequerra E.B., Goyal R., Castro P.A., Levin J.B., Borodinsky L.N. (2018). NMDA Receptor Signaling Is Important for Neural Tube Formation and for Preventing Antiepileptic Drug-Induced Neural Tube Defects NMDA Receptor Signaling Is Important for Neural Tube Formation and for Preventing Antiepileptic Drug-Induced Neural Tube Defec. J. Neurosci..

[71] Borodinsky L.N. (2017). Xenopus Laevis as a Model Organism for the Study of Spinal Cord Formation, Development, Function and Regeneration. Front. Neural Circuits.

[72] Parker S.E., Mai C.T., Canfield M.A., Rickard R., Wang Y., Meyer R.E., Anderson P., Mason C.A., Collins J.S., Kirby R.S. (2010). Updated National Birth Prevalence Estimates for Selected Birth Defects in the United States, 2004–2006. Birth Defects Res. Part A—Clin. Mol. Teratol..

[73] Borodinsky L.N., Belgacem Y.H. (2016). Crosstalk among Electrical Activity, Trophic Factors and Morphogenetic Proteins in the Regulation of Neurotransmitter Phenotype Specification. J. Chem. Neuroanat..

[74] Spitzer N.C. (2015). Neurotransmitter Switching? No Surprise. Neuron.

[75] Spitzer N.C., Debaca R.C., Allen K.A., Holliday J. (1993). Calcium Dependence of Differentiation of GABA Immunoreactivity in Spinal Neurons. J. Comp. Neurol..

[76] Lewis B.B., Miller L.E., Herbst W.A., Saha M.S. (2014). The Role of Voltage-Gated Calcium Channels in Neurotransmitter Phenotype Specification: Coexpression and Functional Analysis in Xenopus Laevis. J. Comp. Neurol..

[77] Borodinsky L.N., Root C.M., Cronin J.A., Sann S.B., Gu X., Spitzer N.C. (2004). Activity-Dependent Homeostatic Specification of Transmitter Expression in Embryonic Neurons. Nature.

[78] Marek K.W., Kurtz L.M., Spitzer N.C. (2010). CJun Integrates Calcium Activity and Tlx3 Expression to Regulate Neurotransmitter Specification. Nat. Neurosci..

[79] Nelson S.B., Valakh V. (2015). Excitatory/Inhibitory Balance and Circuit Homeostasis in Autism Spectrum Disorders. Neuron.

[80] Olmos-Serrano J.L., Paluszkiewicz S.M., Martin B.S., Kaufmann W.E., Corbin J.G., Huntsman M.M. (2010). Defective GABAergic Neurotransmission and Pharmacological Rescue of Neuronal Hyperexcitability in the Amygdala in a Mouse Model of Fragile X. Syndrome. J. Neurosci..

[81] Cellot G., Cherubini E. (2014). GABAergic Signaling as Therapeutic Target for Autism Spectrum Disorders. Front. Pediatr..

[82] Nanou E., Catterall W.A. (2018). Calcium Channels, Synaptic Plasticity, and Neuropsychiatric Disease. Neuron.

[83] Jungbluth H., Treves S., Zorzato F., Sarkozy A., Ochala J., Sewry C., Phadke R., Gautel M., Muntoni F. (2018). Congenital Myopathies: Disorders of Excitation-Contraction Coupling and Muscle Contraction. Nat. Rev. Neurol..

[84] Agrawal A., Suryakumar G., Rathor R. (2018). Role of Defective Ca^2+^ signaling in Skeletal Muscle Weakness: Pharmacological Implications. J. Cell Commun. Signal..

[85] Chernoff E.A.G., Hilfer S.R. (1982). Calcium Dependence and Contraction in Somite Formation. Tissue Cell.

[86] Ferrari M.B., Spitzer N.C. (1999). Calcium Signaling in the Developing *Xenopus* Myotome. Dev. Biol..

[87] Cheung C.Y., Webb S.E., Love D.R., Miller A.L. (2011). Visualization, Characterization and Modulation of Calcium Signaling during the Development of Slow Muscle Cells in Intact Zebrafish Embryos. Int. J. Dev. Biol..

[88] Kelu J.J., Webb S.E., Parrington J., Galione A., Miller A.L. (2017). Ca^2+^ release via Two-Pore Channel Type 2 (TPC2) Is Required for Slow Muscle Cell Myofibrillogenesis and Myotomal Patterning in Intact Zebrafish Embryos. Dev. Biol..

[89] Kelu J.J., Chan H.L.H., Webb S.E., Cheng A.H.H., Ruas M., Parrington J., Galione A., Miller A.L. (2015). Two-Pore Channel 2 Activity Is Required for Slow Muscle Cell-Generated Ca^2+^ Signaling during Myogenesis in Intact Zebrafish. Int. J. Dev. Biol..

[90] Ferrari M.B., Rohrbough J., Spitzer N.C. (1996). Spontaneous Calcium Transients Regulate Myofibrillogenesis in Embryonic Xenopus Myocytes. Dev. Biol..

[91] Campbell N.R., Podugu S.P., Ferrari M.B. (2006). Spatiotemporal Characterization of Short versus Long Duration Calcium Transients in Embryonic Muscle and Their Role in Myofibrillogenesis. Dev. Biol..

[92] Zvaritch E., Depreux F., Kraeva N., Loy R.E., Goonasekera S.A., Boncompagni S., Kraev A., Gramolini A.O., Dirksen R.T., Franzini-Armstrong C. (2007). An Ryr1I4895T Mutation Abolishes Ca^2+^ Release Channel Function and Delays Development in Homozygous Offspring of a Mutant Mouse Line. Proc. Natl. Acad. Sci. USA.

[93] Farini A., Sitzia C., Cassinelli L., Colleoni F., Parolini D., Giovanella U., Maciotta S., Colombo A., Meregalli M., Torrente Y. (2016). Inositol 1,4,5-Trisphosphate (IP3)-Dependent Ca^2+^ Signaling Mediates Delayed Myogenesis in Duchenne Muscular Dystrophy Fetal Muscle. Development.

[94] Allard B. (2018). From Excitation to Intracellular Ca^2+^ movements in Skeletal Muscle: Basic Aspects and Related Clinical Disorders. Neuromuscul. Disord..

[95] Stiber J.A., Rosenberg P.B. (2011). The Role of Store-Operated Calcium Influx in Skeletal Muscle Signaling. Cell Calcium.

[96] McCarl C.A., Picard C., Khalil S., Kawasaki T., Röther J., Papolos A., Kutok J., Hivroz C., LeDeist F., Plogmann K. (2009). ORAI1 Deficiency and Lack of Store-Operated Ca^2+^ entry Cause Immunodeficiency, Myopathy, and Ectodermal Dysplasia. J. Allergy Clin. Immunol..

[97] Picard C., McCarl C.-A., Papolos A., Khalil S., Lüthy K., Hivroz C., LeDeist F., Rieux-Laucat F., Rechavi G., Rao A. (2009). STIM1 Mutation Associated with a Syndrome of Immunodeficiency and Autoimmunity. N. Engl. J. Med..

[98] Pisaniello A. (2003). The Block of Ryanodine Receptors Selectively Inhibits Fetal Myoblast Differentiation. J. Cell Sci..

[99] Webb S.E., Cheung C.C.Y., Chan C.M., Love D.R., Miller A.L. (2012). Application of Complementary Luminescent and Fluorescent Imaging Techniques to Visualize Nuclear and Cytoplasmic Ca^2+^ Signalling during the In Vivo Differentiation of Slow Muscle Cells in Zebrafish Embryos under Normal and Dystrophic Conditions. Clin. Exp. Pharmacol. Physiol..

[100] Kloesel B., Dinardo J.A., Body S.C. (2016). Cardiac Embryology and Molecular Mechanisms of Congenital Heart Disease: A Primer for Anesthesiologists. Anesthesia Analgesia.

[101] Schleich J.M., Abdulla T., Summers R., Houyel L. (2013). An Overview of Cardiac Morphogenesis. Arch. Cardiovasc. Dis..

[102] Moorman A., Webb S., Brown N., Lamers W., Anderson R. (2003). Development of the Heart: Formation of the Cardiac Chambers and Arterial Trunks. Heart.

[103] Créton R., Speksnijder J.E., Jaffe L.F. (1998). Patterns of Free Calcium in Zebrafish Embryos. J. Cell Sci..

[104] Tallini Y.N., Ohkura M., Choi B.-R., Ji G., Imoto K., Doran R., Lee J., Plan P., Wilson J., Xin H.-B. (2006). Imaging Cellular Signals in the Heart in Vivo: Cardiac Expression of the High-Signal Ca^2+^ Indicator GCaMP2. Proc. Natl. Acad. Sci. USA.

[105] Porter G.A., Makuck R.F., Rivkees S.A. (2003). Intracellular Calcium Plays an Essential Role in Cardiac Development. Dev. Dyn..

[106] Hotchkiss A., Feridooni T., Zhang F., Pasumarthi K.B.S. (2014). The Effects of Calcium Channel Blockade on Proliferation and Differentiation of Cardiac Progenitor Cells. Cell Calcium.

[107] Webb S.E., Miller A.L. (2003). Calcium Signalling during Embryonic Development. Nat. Rev. Mol. Cell Biol..

[108] Harvey R.P. (2002). Patterning the Vertebrate Heart. Nat. Rev. Genet..

[109] Carroll T.J., Vize P.D. (1999). Synergism between Pax-8 and Lim-1 in Embryonic Kidney Development. Dev. Biol..

[110] Buisson I., Le Bouffant R., Futel M., Riou J.F., Umbhauer M. (2015). Pax8 and Pax2 Are Specifically Required at Different Steps of Xenopus Pronephros Development. Dev. Biol..

[111] Tena J.J., Neto A., de la Calle-Mustienes E., Bras-Pereira C., Casares F., Gómez-Skarmeta J.L. (2007). Odd-Skipped Genes Encode Repressors That Control Kidney Development. Dev. Biol..

[112] Leclerc C., Webb S.E., Miller A.L., Moreau M. (2008). An Increase in Intracellular Ca^2+^ is Involved in Pronephric Tubule Differentiation in the Amphibian Xenopus Laevis. Dev. Biol..

[113] Gallagher A.R., Hidaka S., Gretz N., Witzgall R. (2002). Molecular Basis of Autosomal-Dominant Polycystic Kidney Disease. Cell. Mol. Life Sci..

[114] Boucek M.M., Snyderman R. (1976). Calcium Influx Requirement for Human Neutrophil Chemotaxis: Inhibition by Lanthanum Chloride. Science.

[115] Oh-hora M., Rao A. (2008). Calcium Signaling in Lymphocytes. Curr. Opin. Immunol..

[116] Oh-Hora M. (2009). Calcium Signaling in the Development and Function of T-Lineage Cells. Immunol. Rev..

[117] Beerman R.W.W., Matty M.A.A., Au G.G.G., Looger L.L.L., Choudhury K.R.R., Keller P.J.J., Tobin D.M.M. (2015). Direct In Vivo Manipulation and Imaging of Calcium Transients in Neutrophils Identify a Critical Role for Leading-Edge Calcium Flux. Cell Rep..

[118] Marks P.W., Maxfield F.R. (1990). Transient Increases in Cytosolic Free Calcium Appear to Be Required for the Migration of Adherent Human Neutrophils. J. Cell Biol..

[119] Hogan P.G., Lewis R.S., Rao A. (2010). Molecular Basis of Calcium Signaling in Lymphocytes: STIM and ORAI. Annu. Rev. Immunol..

[120] Baba Y., Nishida K., Fujii Y., Hirano T., Hikida M., Kurosaki T. (2008). Essential Function for the Calcium Sensor STIM1 in Mast Cell Activation and Anaphylactic Responses. Nat. Immunol..

[121] Vig M., DeHaven W.I., Bird G.S., Billingsley J.M., Wang H., Rao P.E., Hutchings A.B., Jouvin M.-H., Putney J.W., Kinet J.-P. (2008). Defective Mast Cell Effector Functions in Mice Lacking the CRACM1 Pore Subunit of Store-Operated Calcium Release–activated Calcium Channels. Nat. Immunol..

[122] Degen K.E., Gourdie R.G. (2012). Embryonic Wound Healing: A Primer for Engineering Novel Therapies for Tissue Repair. Birth Defects Res. Part C—Embryo Today Rev..

[123] Ud-Din S., Volk S.W., Bayat A. (2014). Regenerative Healing, Scar-Free Healing and Scar Formation across the Species: Current Concepts and Future Perspectives. Exp. Dermatol..

[124] Herrgen L., Voss O.P., Akerman C.J. (2014). Calcium-Dependent Neuroepithelial Contractions Expel Damaged Cells from the Developing Brain. Dev. Cell.

[125] Cordeiro J.V., Jacinto A. (2013). The Role of Transcription-Independent Damage Signals in the Initiation of Epithelial Wound Healing. Nat. Rev. Mol. Cell Biol..

[126] Razzell W., Evans I.R., Martin P., Wood W. (2013). Calcium Flashes Orchestrate the Wound Inflammatory Response through Duox Activation and Hydrogen Peroxide Release. Curr. Biol..

[127] Niethammer P., Grabher C., Look A.T., Mitchison T.J. (2009). A Tissue-Scale Gradient of Hydrogen Peroxide Mediates Rapid Wound Detection in Zebrafish. Nature.

[128] Tu M.K., Borodinsky L.N. (2014). Spontaneous Calcium Transients Manifest in the Regenerating Muscle and Are Necessary for Skeletal Muscle Replenishment. Cell Calcium.

[129] Etulain J. (2018). Platelets in Wound Healing and Regenerative Medicine. Platelets.

[130] Wolf K., Braun A., Haining E.J., Tseng Y.-L., Kraft P., Schuhmann M.K., Gotru S.K., Chen W., Hermanns H.M., Stoll G. (2016). Partially Defective Store Operated Calcium Entry and Hem(ITAM) Signaling in Platelets of Serotonin Transporter Deficient Mice. PLoS ONE.

[131] Chen W., Thielmann I., Gupta S., Subramanian H., Stegner D., van Kruchten R., Dietrich A., Gambaryan S., Heemskerk J.W.M., Hermanns H.M. (2014). Orai1-Induced Store-Operated Ca ^2+^ Entry Enhances Phospholipase Activity and Modulates Canonical Transient Receptor Potential Channel 6 Function in Murine Platelets. J. Thromb. Haemost..

[132] Gotru S.K., Chen W., Kraft P., Becker I.C., Wolf K., Stritt S., Zierler S., Hermanns H.M., Rao D., Perraud A.L. (2018). TRPM7 Kinase Controls Calcium Responses in Arterial Thrombosis and Stroke in Mice. Arterioscler. Thromb. Vasc. Biol..

[133] Lysaght M.J., Jaklenec A., Deweerd E. (2008). Great Expectations: Private Sector Activity in Tissue Engineering, Regenerative Medicine, and Stem Cell Therapeutics. Tissue Eng. Part A.

[134] Patterson R.A., Juarez M.T., Hermann A., Sasik R., Hardiman G., McGinnis W. (2013). Serine Proteolytic Pathway Activation Reveals an Expanded Ensemble of Wound Response Genes in Drosophila. PLoS ONE.

[135] Tsujioka H., Kunieda T., Katou Y., Shirahige K., Kubo T. (2015). Unique Gene Expression Profile of the Proliferating Xenopus Tadpole Tail Blastema Cells Deciphered by RNA-Sequencing Analysis. PLoS ONE.

[136] Mouse Gene Expression Data Search. http://www.informatics.jax.org/gxd.

[137] ZFIN Expression Search. https://zfin.org/action/expression/search.

[138] Gene Expression Search. http://www.xenbase.org/geneExpression/geneExpressionSearch.do?method=display.

[139] Kolios G., Moodley Y. (2013). Introduction to Stem Cells and Regenerative Medicine. Respiration.

[140] Ermakov A., Daks A., Fedorova O., Shuvalov O., Barlev N.A. (2018). Ca^2+^-Depended Signaling Pathways Regulate Self-Renewal and Pluripotency of Stem Cells. Cell Biol. Int..

[141] Rahaman M.N., Mao J.J. (2005). Stem Cell-Based Composite Tissue Constructs for Regenerative Medicine. Biotechnol. Bioeng..

[142] Hasan A. (2016). Tissue Engineering for Artificial Organs: Regenerative Medicine, Smart Diagnostics and Personalized Medicine.

[143] Jergova S., Gajavelli S., Varghese M.S., Shekane P., Sagen J. (2016). Analgesic Effect of Recombinant GABAergic Cells in a Model of Peripheral Neuropathic Pain. Cell Transplant..

[144] Méry A., Aimond F., Ménard C., Mikoshiba K., Michalak M., Pucéat M. (2005). Initiation of Embryonic Cardiac Pacemaker Activity by Inositol 1,4,5-Trisphosphate-Dependent Calcium Signaling. Mol. Biol. Cell.

[145] Limpitikul W.B., Dick I.E., Tester D.J., Boczek N.J., Limphong P., Yang W., Choi M.H., Babich J., Disilvestre D., Kanter R.J. (2017). A Precision Medicine Approach to the Rescue of Function on Malignant Calmodulinopathic Long-QT Syndrome. Circ. Res..

