# Supplementary material for: Calcium Signaling in Vertebrate Development and Its Role in Disease
Source: Int J Mol Sci. 2018 Oct 30;19(11):3390. doi: 10.3390/ijms19113390 (PMC6274931; doi:10.3390/ijms19113390)
Supplement: Supplementary file 1 [file ijms-19-03390-s001.pdf]

Supplementary table 1. Common diseases associated with calcium channels and pumps, and their comparative embryonic expression patterns in mouse, zebrafish and *Xenopus* [1,136–138].

Gene Symbol	Gene Name	Diseases/Disorders	Embryonic Expression in Mouse	Embryonic Expression in Zebrafish	Embryonic Expression in *Xenopus*
Cacna1a	Calcium channel, voltage-dependent, P/Q type, alpha 1A subunit	Spinocerebellar ataxia 6 (SCA6) [MIM:183086], Migraine, familial hemiplegic, 1 (FHM1) [MIM:141500], Episodic ataxia 2 (EA2) [MIM:108500], Epileptic encephalopathy, early infantile, 42 (EIEE42) [MIM:617106]	Earliest stage of expression is E11.5 , and this gene is expressed in the following tissues: Brain	Expression reported from 50% epiboly to Day 5 in following tissues: Central nervous system, cranial ganglion, neuron, retinal ganglion cell layer, Rohon-Beard neuron, spinal cord dorsal region, whole organism	Earliest stage of expression detected using ISH is stages 13 , and this gene is expressed in the following tissues: Auditory nerve, cranial placode, eye, facial nerve, forebrain, glossopharyngeal nerve, hindbrain, lens, midbrain, neural tube, pineal gland, retinal ganglion cell layer, retinal inner nuclear layer
Cacna1b	Calcium channel, voltage-dependent, N type, alpha 1B subunit	Dystonia 23 (DYT23) [MIM:614860]	Earliest stage of expression is E8.25 , and this gene is expressed in the following tissues: Embryo endoderm, brain and retina	Expression reported from day 5 to adult in brain, liver and whole organism	Earliest stage of expression detected using ISH is stages 13 , and this gene is expressed in the following tissues: Auditory nerve, brain, cranial placode, eye, facial nerve, forebrain, glossopharyngeal nerve, hindbrain, lens, midbrain, neural tube, retinal ganglion cell layer, retinal inner nuclear layer, spinal c
Cacna1c	Calcium channel, voltage-dependent, L type, alpha 1C subunit	Timothy syndrome (TS) [MIM:601005], Brugada syndrome 3 (BRGDA3) [MIM:611875]	Earliest stage of expression is E8.5 , and this gene is expressed in the following tissues: Heart	Expression reported from 20-25 somites to Day 5 in Central nervous system, ceratohyal cartilage, gut, lateral plate mesoderm	Earliest stage of expression detected using ISH is stages 13 , and this gene is expressed in the following tissues: Auditory nerve, brain, cranial ganglion, cranial placode, eye, facial nerve, forebrain, fused heart primordium, glossopharyngeal nerve, heart, hindbrain, lens, midbrain, neural tube, photoreceptor lay

Cacna1d	Calcium channel, voltage-dependent, L type, alpha 1D subunit	Sinoatrial node dysfunction and deafness (SANDD) [MIM:614896], Primary aldosteronism, seizures, and neurologic abnormalities (PASNA) [MIM:615474],	Earliest stage of expression is E9.5 , and this gene is expressed in the following tissues: Heart	Expression reported from 20-cell to day 6 in anterior lateral line neuromast neuromast hair cell, central nervous system, neuromast hair cell presynaptic active zone, posterior lateral line neuromast neuromast hair cell	Earliest stage of expression detected using ISH is stages 24 , and this gene is expressed in the following tissues: Brain, eye, facial nerve, forebrain, fused heart primordium, heart, hindbrain, midbrain, otic placode, otic vesicle, photoreceptor layer, pineal gland, spinal cord, trigeminal nerve
Cacna1e	Calcium channel, voltage-dependent, R type, alpha 1E subunit	No disorders are reported	Earliest stage of expression is E14.5, and this gene is expressed in the following tissues: Thalamus mantle layer, Cerebral and olfactory cortex mantle layer, telencephalon mantle layer, medulla oblongata basal plate mantle layer, pons mantle and trigeminal ganglion	Expression reported from day 5 in whole organism	Earliest stage of expression detected using ISH is stages 24 , and this gene is expressed in the following tissues: Hindbrain, spinal cord
Cacna1f	Calcium channel, voltage-dependent, L type, alpha 1F subunit	Night blindness, congenital stationary, 2A (CSNB2A) [MIM:300071], Cone-rod dystrophy, X-linked 3 (CORDX3) [MIM:300476], Aaland island eye disease (AIED) [MIM:300600]		Expression reported from day 5 in whole organism Protruding-mouth to adult in photoreceptor cell synapse, retinal outer plexiform layer, retinal photoreceptor layer	Earliest stage of expression detected using ISH is stages 34 , and this gene is expressed in the following tissues: Eye, lens, retina, retinal ganglion cell layer, retinal inner nuclear layer
Cacna1g	Calcium channel, voltage-dependent, T type, alpha 1G subunit	Spinocerebellar ataxia 42 (SCA42) [MIM:616795]:	Earliest stage of expression is E8.5 , and this gene is expressed in the following tissues: Brain, diencephalon, telencephalon, future spinal cord, primitive venritlce, hindlimb musculature, heart	75%-epiboly to day 5 in whole organism	Earliest stage of expression detected using ISH is stages 30 , and this gene is expressed in the following tissues: Brain, eye, facial nerve, forebrain, hindbrain, midbrain, olfactory epithelial support cell, otic placode, otic vesicle, pineal gland, spinal cord, trigeminal nerve

Cacna1h	Calcium channel, voltage-dependent, T type, alpha 1H subunit	Epilepsy, idiopathic generalized 6 (EIG6) [MIM:611942], Epilepsy, childhood absence 6 (ECA6) [MIM:611942], Hyperaldosteronism, familial, 4 (HALD4) [MIM:617027]	Earliest stage of expression is E8.5, and this gene is expressed in the following tissues: Neural tube floor plate, future spinal cord, dorsal root ganglion, brain, spinal cord floor plate, heart	50%-epiboly to Pec-fin in adaxial cell, diencephalon, retinal ganglion cell layer, retinal inner nuclear layer	Earliest stage of expression detected using ISH is stages 13 , and this gene is expressed in the following tissues: Brain, eye, forebrain, fused heart primordium, heart, hindbrain, midbrain, neural tube, olfactory epithelial support cell, spinal cord
Cacna1i	Calcium channel, voltage-dependent, T type, alpha 1I subunit	Childhood Absence Epilepsy	Earliest stage of expression is E11.5, and this gene is expressed in the following tissues: primitive ventricle, hindlimb musculature	75%-epiboly to day 5 in whole organism	-
Cacna1s	Calcium channel, voltage-dependent, L type, alpha 1S subunit	Periodic paralysis hypokalemic 1 (HOKPP1) [MIM:170400], Malignant hyperthermia 5 (MHS5) [MIM:601887, Thyrotoxic periodic paralysis 1 (TTPP1) [MIM:188580]	Earliest stage of expression is E9.5 , and this gene is expressed in the following tissues: Hindbrain, diencephalon lateral wall mantle layer, medulla oblongata basal plate mantle layer, cerebellum mantle layer, pons,	Expression reported from 20-25 somites stage	Earliest stage of expression detected using ISH is stages 7 , and this gene is expressed in the following tissues: Animal hemisphere, dorsal, dorsal marginal zone, ectoderm, epaxial muscle, mesoderm, muscle, musculature of face, paraxial mesoderm, pharyngeal arch, presomitic mesoderm, somite, tail somite
Ryr1	ryanodine receptor 1, skeletal muscle	Malignant hyperthermia 1 (MHS1) [MIM:145600], Central core disease of muscle (CCD) [MIM:117000], Multiminicore disease with external ophthalmoplegia (MMDO) [MIM:255320]	Earliest stage of expression is E9.5 , and this gene is expressed in the following tissues: Diencephalon, telencephalon, hindbrain, future spinal cord, heart	Expression reported from 1-cell to adult in adaxial cell, fast muscle cell, heart, slow muscle cell, slow muscle cell, somite, trunk	Earliest stage of expression detected using ISH is stages 30 , and this gene is expressed in the following tissues: Epaxial muscle, muscle, myoblast, skeletal muscle

Ryr2	Ryanodine receptor 2, cardiac	Arrhythmogenic right ventricular dysplasia, familial, 2 (ARVD2) [MIM:600996], Ventricular tachycardia, catecholaminergic polymorphic, 1, with or without atrial dysfunction and/or dilated cardiomyopathy (CPVT1) [MIM:604772]	Earliest stage of expression is E7.5 , and this gene is expressed in the following tissues: Myotome, cerebral cortex, tongue, foot, messeter, diaphragm, telencephalon, skeletal muscle	Expression reported from 4-cell to adult in cardiac muscle, heart, heart primordium, heart tube, brain	-
Ryr3	Ryanodine receptor 3	arrhythmogenic right ventricular dysplasia, familial, 2, central core myopathy and alzheimer disease	Earliest stage of expression is E15, and this gene is expressed in the following tissues: Heart, somite, liver,	Expression reported from 4-cell to adult in brain, fast muscle cell, habenula, hindbrain	-
Orai1	ORAI calcium release-activated calcium modulator 1	Immunodeficiency 9 (IMD9) [MIM:612782], Myopathy, tubular aggregate, 2 (TAM2) [MIM:615883]	Earliest stage of expression is E14, and this gene is expressed in the following tissues: Embryonic brain and limb musculature, other organs including, heart, liver, brain and metanephros	Expression reported from 1-cell to Pec-fin in Brain heart liver myotome	-
Orai2	ORAI calcium release-activated calcium modulator 2	No disorders are reported	Earliest stage of expression is E14.5, and this gene is expressed in the following tissues: Neural tissue, brian including trigeminal ganglion, vagus ganglion, olfactory epithelium, nasal cavity respiratory epithelium	-	Earliest stage of expression detected using ISH is stages 10 , and this gene is expressed in the following tissues: Dorsal regions
Orai3	ORAI calcium release-activated calcium modulator 3	No disorders are reported	Earliest stage of expression is , and this gene is expressed in the following tissues: Brain, trigeminal ganglion, neural retina, naris, nasal cavity respiratiory epithelium, vibrissa, jaw, vestibulo-cochlear ganglion, vestibulo-cpclear ganglion and spinal cord	-	-

P2ry1	Purinergic receptor P2Y, G-protein coupled, 1	renal cell carcinoma, nonpapillary, clopidogrel resistance		-	Earliest stage of expression detected using ISH is stages 1 , and this gene is expressed in the following tissues: Muscle
P2ry2	Purinergic receptor P2Y, G-protein coupled, 2	dry eye syndrome, lacrimal apparatus disease, cystic fibrosis		-	-
P2ry3	Purinergic receptor P2Y, G-protein coupled, 3	Non-ammalian	Earliest stage of expression is , and this gene is expressed in the following tissues:	-	-
P2ry4	pyrimidinergic receptor P2Y, G-protein coupled, 4	No disorders are reported		-	Earliest stage of expression detected using ISH is stages 14 , and this gene is expressed in the following tissues: Anterior neural ridge, chordal neural plate, cranial neural crest, neural crest, neural plate, neural tube, posterior neural tube, pre-chordal neural plate, tail bud, cranial neural crest, mandibular arch, neural crest, neural plate border
P2ry5	Purinergic receptor P2Y, G-Protein Coupled, 5. New name- Lysophosphatidic Acid Receptor 6 (LPAR6)	Woolly hair autosomal recessive 1 with or without hypotrichosis (ARWH1) [MIM:278150], Hypotrichosis 8 (HYPT8) [MIM:278150]	Earliest stage of expression is E1, and this gene is expressed in the following tissues: Skeletal muscle	-	-
P2ry6	pyrimidinergic receptor P2Y, G-protein coupled, 6	No disorders are reported	Earliest stage of expression is E9, and this gene is expressed in the following tissues: Blastocyst-stage conceptus, foregut	-	-
P2ry7	Purinergic receptor P2Y, G-Protein Coupled, 7, New name- Leukotriene B4 Receptor	specific language impairment, asthma	Earliest stage of expression is E15, and this gene is expressed in the following tissues: Dermomyotome, muscle tissue, vertebral axis, musculature	-	-

P2ry8	P2Y receptor family member 8	Non-ammalian	Earliest stage of expression is , and this gene is expressed in the following tissues: Lateral ventricle and brain	-	-
P2ry9	P2Y receptor family member 9, New name- Lysophosphatidic Acid Receptor 4	differentiated thyroid carcinoma		-	-
P2ry10	P2Y receptor family member 10	No disorders are reported	Earliest stage of expression is E14.5, and this gene is expressed in the following tissues: Future brain, including future midbrain and future hindbrain, 1st branchial arch maxillary component, somite, liver	-	-
P2ry11	Purinergic receptor P2Y, G-protein coupled, 11	Narcolepsy	Earliest stage of expression is , and this gene is expressed in the following tissues: Thymus primordium	-	Earliest stage of expression detected using ISH is stages 11 , and this gene is expressed in the following tissues: Mesoderm and notochord
P2ry12	Purinergic receptor P2Y, G-protein coupled, 12	Bleeding disorder, platelet-type 8 (BDPLT8) [MIM:609821], coronary heart disease		-	
P2ry13	Purinergic receptor P2Y, G-protein coupled, 13	No disorders are reported		-	
P2ry14	Purinergic receptor P2Y14	No disorders are reported		-	
Trpc1	transient receptor potential cation channel, subfamily C, member 1	Pulmonary hypertension	Earliest stage of expression is E12.0 , and this gene is expressed in the following tissues: Vagus ganglion, DRG	Expression reported from adult in Brain, bulbus arteriosus, cranial ganglion, diencephalon	Earliest stage of expression detected using ISH is stages 20 , and this gene is expressed in the following tissues: Brain, central nervous system, cranial nerve, profundal placode, spinal cord, trigeminal nerve, trigeminal placode

Trpc2	transient receptor potential cation channel, subfamily C, member 2	No disorders are reported	Earliest stage of expression is E12.5 , and this gene is expressed in the following tissues: Metanephros, ovary, testis, mesonephros of female, reproductive system, urogenital mesentery, DRG, vagus ganglion, primitive bladder	Expression reported from Prim-5 to adult in microvillous olfactory receptor neuron, olfactory epithelium, olfactory receptor cell, olfactory rosette	Earliest stage of expression detected using ISH is stages 28 , and this gene is expressed in the following tissues: Brain, central nervous system, eye, hindbrain, melanophore, neural crest, optic vesicle, otic vesicle, spinal cord
Trpc3	transient receptor potential cation channel, subfamily C, member 3	Spinocerebellar ataxia 41 (SCA41) [MIM:616410]	Earliest stage of expression is E11.5 , and this gene is expressed in the following tissues: Limb, trunk somite, vagus ganglion, DRG, vagus ganglion	Expression reported from Prim-5 to Day 5 in cerebellum, diencephalon, dorsal telencephalon, hindbrain	
Trpc4	transient receptor potential cation channel, subfamily C, member 4	No disorders are reported	Earliest stage of expression is E11.5 , and this gene is expressed in the following tissues: Brain, trunk somite, brain stem, cerebral cortex, cerebellum, vagus ganglion, DRG	Expression reported from Prim-5 to adult in cranial ganglion, dorsal telencephalon, epibranchial ganglion, glossopharyngeal ganglion	
Trpc5	transient receptor potential cation channel, subfamily C, member 5	congenital hypertrophic pyloric stenosis, hypertensive disease	Earliest stage of expression is E11.5 , and this gene is expressed in the following tissues: Brain, vagus ganglion, DRG	Expression reported from Prim-5 to adult in brain, habenula, head, hindBrain	
Trpc6	transient receptor potential cation channel, subfamily C, member 6	Congenital nephrotic syndrome, Focal segmental glomerulosclerosis 2 (FSGS2) [MIM:603965]	Earliest stage of expression is E12.0 , and this gene is expressed in the following tissues: Vagus ganglion, DRG	Expression reported from to adult in trigeminal motor nucleus, cardiac ventricle, cloacal chamber, dorsal aorta and gut	
Trpv1	transient receptor potential cation channel, subfamily V, member 1	somatoform disorder, urinary bladder disease	Earliest stage of expression is E11.5 , and this gene is expressed in the following tissues: Diencephalon, telencephalon, hindbrain, future spinal cord	Expression reported from 4-cell to adult in anterior lateral line ganglion, brain, epidermal cell and epithelium	Earliest stage of expression detected using ISH is stages 20 , and this gene is expressed in the following tissues: Trigeminal ganglia, dorsal neural tube, Rohon Beard neurons and somites

Trpv2	transient receptor potential cation channel, subfamily V, member 2	dentin sensitivity, ymphoma of retina	Earliest stage of expression is E14.5, and this gene is expressed in the following tissues: Future brain, including future midbrain and future hindbrain, 1st branchial arch maxillary component, somite, liver	-	Earliest stage of expression detected using ISH is stages 1 , and this gene is expressed in the following tissues: Animal pole, and in the cement gland, neural tube, trigeminal ganglia and otic vesicle at later stages of development
Trpv3	transient receptor potential cation channel, subfamily V, member 3	Olmsted syndrome (OLMS) [MIM:614594], Palmoplantar keratoderma, non-epidermolytic, focal 2 (FNEPPK2) [MIM:616400]	Earliest stage of expression is E11.5, and this gene is expressed in the following tissues: Diencephalon, telencephalon, hindbrain, futurespinal cord	-	Trpv3 is not detected using ISH and RT-PCR throughout embryonic stages.
Trpv4	transient receptor potential cation channel, subfamily V, member 4	Brachyolmia 3 (BCYM3) [MIM:113500], Spondylometaphyseal dysplasia Kozlowski type (SMDK) [MIM:184252], Metatropic dysplasia (MTD) [MIM:156530], Neuronopathy, distal hereditary motor, 8 (HMN8) [MIM:600175], Charcot-Marie-Tooth disease 2C (CMT2C) [MIM:606071]	Earliest stage of expression is E11.5 , and this gene is expressed in the following tissues: Diencephalon, telencephalon, hindbrain, futurespinal cord	Expression reported from 4-cell to adult in atrioventricular canal atrioventricular canal endocardium endothelial cell atrioventricular canal endocardium atrioventricular canal morphogenesis	Earliest stage of expression detected using ISH is stages 1 , and this gene is expressed in the following tissues: Animal pole, and in the tailbud region and notochord and nasal placode at later stages of development.
Trpv5	transient receptor potential cation channel, subfamily V, member 5	Pseudohermaphroditism, female, due to placental aromatase deficiency	Earliest stage of expression is E15.5, and this gene is expressed in the following tissues: Hypothalamus, neocortex, ganglionic eminence, main olfactory bulb, spinal cord	-	Earliest stage of expression detected using ISH is stages 15, and this gene is expressed in the following tissues: Proctodeum, branchial arches and punctate expression in epidermis.

Trpv6	transient receptor potential cation channel, subfamily V, member 6	Pseudohermaphroditism, female, due to placental aromatase deficiency	Earliest stage of expression is E11.5 , and this gene is expressed in the following tissues: Diencephalon, hindbrain, midbrain, future spinal cord, stomach epithelium, metanephros, stomach	Expression reported from 1-cell to adult in amacrine cell, anterior crista hair cell, atrium and blastomere	Earliest stage of expression detected using ISH is stages 15, and this gene is expressed in the following tissues: Epidermis.
Trpm1	transient receptor potential cation channel, subfamily M, member 1	Night blindness, congenital stationary, 1C (CSNB1C) [MIM:613216]	Earliest stage of expression is E11.5 , and this gene is expressed in the following tissues: Eye, pigmented retinal epithelium, genital tubercle, reproductive system, retina	Expression reported from Prim-5 to adult in optic furrow, presumptive retinal pigmented epithelium, retinal inner nuclear layer, retinal pigmented epithelium, ciliary marginal zone, melanoblast, neural crest cell, and retina	
Trpm2	transient receptor potential cation channel, subfamily M, member 2	No disorders are reported	Earliest stage of expression is E10.5 , and this gene is expressed in the following tissues: Central nervous system including choroid plexus, cranial ganglion, ear, eye, face, gut, heart, limb, liver, lung, hemolymphoid system, main olfactory bulb, nephros, salivary gland, thyroid gland, urinary system	Expression reported from Prim-5 to adult in cranial ganglion, hindbrain, integument, and keratinocyte	
Trpm3	transient receptor potential cation channel, subfamily M, member 3	Retinal dystrophy and iris coloboma with or without congenital cataract	Earliest stage of expression is E12.0 , and this gene is expressed in the following tissues: Neural retinal epithelium, dorsal root ganglion, inferior vagus ganglion	Expression reported from Prim-5 to adult in choroid plexus, dorsal thalamus, epiphysis, and habenula	
Trpm4	transient receptor potential cation channel, subfamily M, member 4	Progressive familial heart block 1B (PFHB1B) [MIM:604559]	Earliest stage of expression is E12.0 , and this gene is expressed in the following tissues: Inferior vagus ganglion, DRG	Expression reported from 1-cell to adult in Brain, cloacal chamber, integument, and intestinal bulb	

Trpm5	transient receptor potential cation channel, subfamily M, member 5	Beckwith-wiedemann syndrome	Earliest stage of expression is E12.0 , and this gene is expressed in the following tissues: Inferior vagus ganglion, DRG	Expression reported from Protruding-mouth to adult in barbel, gill taste receptor cell, gill, and gill raker	
Trpm6	transient receptor potential cation channel, subfamily M, member 6	Hypomagnesemia 1 (HOMG1), including seizures and muscle spasms, during infancy [MIM:602014]	Earliest stage of expression is E12.0 , and this gene is expressed in the following tissues: Inferior vagus ganglion, DRG	Expression reported from 1-cell to adult in Brain, gill, gut, and heart	Earliest stage of expression detected using ISH is stages 17 , and this gene is expressed in the following tissues: Anterior placodal area, brain, cement gland primordium, endoderm, neural plate border, non-neural ectoderm, notochord, posterior, primary heart field, spinal cord
Trpm7	transient receptor potential cation channel, subfamily M, member 7	Neurodegenerative disorder amyotrophic lateral sclerosis-parkinsonism/dementia complex 1 (ALS-PDC1) [MIM:105500]	Earliest stage of expression is E12.0 , and this gene is expressed in the following tissues: Inferior vagus ganglion, DRG	Expression reported from to adult in corpuscles of Stannius, epidermis, epiphysis, and head	Earliest stage of expression detected using ISH is stages 2 , and this gene is expressed in the following tissues: Rohon-Beard neuron, animal cap, animal pole, axial mesoderm, brain, branchial arch, cardiac mesoderm, head region, intersomitic region, involuted dorsal mesoderm, late distal tubule, neuron, notochord
Trpm8	transient receptor potential cation channel, subfamily M, member 8	Lichen sclerosus et atrophicus, migraine with or without aura 1	Earliest stage of expression is E12.0 , and this gene is expressed in the following tissues: Inferior vagus ganglion, DRG	-	

Stim1	stromal interaction molecule 1	Immunodeficiency 10 (IMD10) [MIM:612783], Myopathy, tubular aggregate, 1 (TAM1) [MIM:160565], Stormorken syndrome (STRMK) [MIM:185070]	Earliest stage of expression is E7.5, and this gene is expressed in the following tissues: Embryo, embryonic brain, heart, liver, gut, urinary system, extraembryonic component	-	Earliest stage of expression detected using ISH is stages 3 , and this gene is expressed in the following tissues: Animal hemisphere, animal pole, cement gland, cement gland primordium, ectoderm, eye, head, head region, involuted dorsal mesoderm, involuted ventral mesoderm, involuting marginal zone, marginal zone,, brain, central nervous system, growth cone, notochord, process, spinal cord, spinal neuron
Stim2	stromal interaction molecule 2	Stormorken syndrome and myopathy, tubular aggregate 1		-	
P2rx1	Purinergic receptor P2X, ligand gated ion channel, 1	Neurogenic dysfunction of the urinary bladder, dilated cardiomyopathy and ctrct3	Earliest stage of expression is E14.5, and this gene is expressed in the following tissues: Pancreatic epithelium	Expression reported from Prim-15 stage in hypothalamus and midbrain	
P2rx2	Purinergic receptor P2X, ligand gated ion channel, 2	Deafness, autosomal dominant, 41 (DFNA41) [MIM:608224], Neurogenic bladder, bladder disease	Earliest stage of expression is E14.5, and this gene is expressed in the following tissues: Medulla oblongata basal plate mantle layer, VG, facial ganglion,	Expression reported from Prim-15 to Pec-fin stage in cranial ganglion, facial ganglion, glossopharyngeal ganglion, and Rohon-Beard neuron	
P2rx3	Purinergic receptor P2X, ligand gated ion channel, 3	Cystitis and migraine with or without aura 1	Earliest stage of expression is E14.5, and this gene is expressed in the following tissues: Medulla oblongata basal plate, facial ganglion, glossopharyngeal ganglion, trigeminal ganglion, vestibulo-cochlear ganglion	Expression reported from Prim-15 to Protruding-mouth stage in CNS neuron (sensu Vertebrata), cranial ganglion, head ectoderm, and Rohon-Beard neuron	
P2rx4	Purinergic receptor P2X, ligand gated ion channel, 4	Chronic pain	Earliest stage of expression is E11.5 , and this gene is expressed in the following tissues: Hindbrain, midbrain, future spinal cord	Expression reported from 1-cell to Pec-fin stage	

P2rx5	Purinergic receptor P2X, ligand gated ion channel, 5	Cystinosis	Earliest stage of expression is E8.5 , and this gene is expressed in the following tissues: Future brain, neural ectoderm, future spinal cord, dorsal root ganglion,	Expression reported from Prim-5 to Prim-15 stage in muscle skeletal, muscle cell and somite	
P2rx6	Purinergic receptor P2X, ligand gated ion channel, 6	No disorders are reported	Earliest stage of expression is E10.5 , and this gene is expressed in the following tissues: Heart	-	
P2rx7	Purinergic receptor P2X, ligand gated ion channel, 7	Extrapulmonary tuberculosis, bone cancer, tularemia and root resorption	Earliest stage of expression is E17.5 , and this gene is expressed in the following tissues: Metanephros, heart, brain	Expression reported from 1-cell to Pec-fin stage in Brain, midBrain hindBrain boundary, pronephric duct and spinal cord	
P2rx8	Purinergic receptor P2X, ligand gated ion channel, 8	No disorders are reported	-	Expression reported from Prim-15 to Pec-fin stage in cranial ganglion, hypothalamus, lateral hypothalamic nucleus and Rohon-Beard neuron	
Itpr1	Inositol 1,4,5-trisphosphate receptor 1	Spinocerebellar ataxia 15 (SCA15) [MIM:606658], Spinocerebellar ataxia 29 (SCA29) [MIM:117360] and Gillespie syndrome (GLSP) [MIM:206700]	Earliest stage of expression is E5.5 , and this gene is expressed in the following tissues: Embryo, primitive streak, mesoderm, neural tube, heart, branchial arch, somite, CNS, alimentary system, pituitary gland, reproductive structure	Expression reported from 1-cell to adult in whole organism, including cerebellum, granule cell, hindbrain and optic tectum	Earliest stage of expression detected using ISH is stages 1 , and this gene is expressed in the following tissues: Cytoplasm, oocyte, subcortical cytoplasm
Itpr2	Inositol 1,4,5-triphosphate receptor 2	Anhidrosis, isolated, with normal sweat glands (ANHD) [MIM:106190] and absence of sweating	Earliest stage of expression is E14.5, and this gene is expressed in the following tissues: Medulla oblongata, appendages, scapula	Expression reported from 64-cell stage in whole organism	

Itpr3	Inositol 1,4,5-triphosphate receptor 3	Alzheimer disease, coronary artery aneurysm, peritoneum cancer, and diabetes mellitus	Earliest stage of expression is E14.5, and this gene is expressed in the following tissues: Appendage bones, basioccipital bone, turbinate bone primordium, vault of skull, orbito-sphenoid, otic capsure, nasal septum, rib, axial skeleton, Meckel's cartilage	Expression reported from 64-cell to protruding-mouth stage in brain, head, heart and heart tube	
Atp2a1	ATPase, Ca2+ transporting, cardiac muscle, fast twitch 1	Brody myopathy (BRM) [MIM:601003], Wolfram syndrome 2 and myotonic disease	Earliest stage of expression is E14.5, and this gene is expressed in the following tissues: Appendage mesenchyme, tail mesenchyme, diaphragm, tongue, foot	Expression reported from 2-cell to adult in adaxial cell, eye, gill and mesoderm	
Atp2a2	ATPase, Ca2+ transporting, cardiac muscle, slow twitch 2	Acrokeratosis verruciformis (AKV) [MIM:101900] and Darier disease (DD) [MIM:124200]	Earliest stage of expression is E14.5 , and this gene is expressed in the following tissues: Pancreas epithelium, interventricular septum, atrioventricular bundle, impulse conducting system	Expression reported from 1-cell to adult in axis, myotome, pectoral fin and pectoral fin musculature, atrium, cardiac ventricle, cephalic musculature and heart	Earliest stage of expression detected using ISH is stages 14 , and this gene is expressed in the following tissues: Branchial arch, cement gland, ectoderm, eye, heart, lens, mesenchyme, neural plate, neural tube, proctodeum, pronephric kidney, retina
Atp2a3	ATPase, Ca2+ transporting, cardiac muscle, slow twitch 3	Darier-white disease and brody myopathy		Expression reported from Day 5 to adult in Purkinje cell	Earliest stage of expression detected using ISH is stages 1 , and this gene is expressed in the following tissues: Cement gland, ectoderm, ectodermal cell, marginal zone, neural plate, neuroectoderm, stage VI oocyte, vegetal, vegetal pole, zygote cortex
Atp2b1	ATPase, Ca2+ transporting, plasma membrane 1	Deafness, autosomal dominant, 25	Earliest stage of expression is E9.5 , and this gene is expressed in the following tissues: CNS, apendages, ear, alimentary system, urinary system, integumental system, yes, lung, pituitary gland		

Atp2b2	ATPase, Ca2+ transporting, plasma membrane 2	Deafness, autosomal recessive 12 and leber congenital amaurosis	Earliest stage of expression is E12.5 , and this gene is expressed in the following tissues: CNS, pituitary gland, DRG, choroid plexus, facial ganglion, glossopharyngeal ganglia, trigeminal ganglia, alfactory cortex, piliform cortex		
Atp2b3	ATPase, Ca2+ transporting, plasma membrane 3	Spinocerebellar ataxia, X-linked 1 (SCAX1) [MIM:302500], and microcephaly	Earliest stage of expression is E12.5 , and this gene is expressed in the following tissues: CNS, ear, alimentary system, spleen primordium, reproductive system, liver		
Atp2b4	ATPase, Ca2+ transporting, plasma membrane 4	Schnyder corneal dystrophy. Long-QT syndrome and malaria susceptibility	Earliest stage of expression is E9.5, and this gene is expressed in the following tissues: Heart, CNS, including ventral gray horn and Spinal cord, and liver		
Atp2c1	ATPase, Ca2+-sequestering	Hailey-Hailey disease (HHD) [MIM:169600], benign chronic pemphigus, darier-white disease and skin disease	Earliest stage of expression is E8.5, and this gene is expressed in the following tissues: Embryo, and adult tissues including, brai, heart, stomach, colon, liver and lung		
Atp2c2	ATPase Secretory Pathway Ca2+ Transporting 2	Dyslexia, darier-white disease and speech and communication disorder			
Atp1a1	ATPase, Na+/K+ transporting, alpha 1 polypeptide	Aldosterone-producing adenoma, thyrotoxic periodic paralysis, endocrine organ benign neoplasm and organ system benign neoplasm	Earliest stage of expression is E9.5 , and this gene is expressed in the following tissues: Heart, nervous system including spinal cord and DRG, limbs, tail, cranium, choroid plaexu, pituitary gland, cardiovascular system		

Atp1a2	ATPase Na+/K+ Transporting Subunit Alpha 2	Migraine, familial hemiplegic, 2 (FHM2) [MIM:602481] and Alternating hemiplegia of childhood 1 (AHC1) [MIM:104290]	Earliest stage of expression is E9.5, and this gene is expressed in the following tissues: Primitive ventricle, heart atrium, myotome, future brain, limbs, reproduction system and tail		
Atp1a3	ATPase, Na+/K+ transporting, alpha 3 polypeptide	Dystonia 12 (DYT12) [MIM:128235], Alternating hemiplegia of childhood 2 (AHC2) [MIM:614820], and Cerebellar ataxia, areflexia, pes cavus, optic atrophy, and sensorineural hearing loss (CAPOS) [MIM:601338]	Earliest stage of expression is E9.5 , and this gene is expressed in the following tissues: Neural tube, telencephalon marginal layer, hindbrain, eye, optic stalk, heart, cranial ganglion, future spinal cord, main olfactory bulb, respiratory system, alimentary system		
Atp1b1	ATPase, Na+/K+ transporting, beta 1 polypeptide	Hemiplegic migraine and fuchs' endothelial dystrophy, and hypertension-essential	Earliest stage of expression is E11.5 , and this gene is expressed in the following tissues: Brain including diencephalon, telencephalon, hindbrain, future spinal cord, colliculus, choroid plexus, pituitary gland, ear, thyroid gland, ureter, respiratory system		
